# Swimmer’s itch in Canada: a look at the past and a survey of the present to plan for the future

**DOI:** 10.1186/s12940-018-0417-7

**Published:** 2018-10-25

**Authors:** Michelle A Gordy, Tyler P Cobb, Patrick C Hanington

**Affiliations:** 1 School of Public Health, 357F South Academic Building, University of Alberta, Edmonton, AB T6G 2G7 Canada; 2 Invertebrate Zoology, Royal Alberta Museum, 9810 103A Ave, Edmonton, AB T5J 0G2 Canada; 3 Alberta Biodiversity Monitoring Institute, CW405 Biological Sciences Building, University of Alberta, Edmonton, AB T6G 2E9 Canada

**Keywords:** Cercarial dermatitis, Swimmer’s itch, Schistosome, Trichobilharzia, Alberta, Host-distributions, Surveillance, Schistosomatium, Canada

## Abstract

**Background:**

Cercarial dermatitis, colloquially “swimmer’s itch”, is a rash contracted in natural bodies of water, when people are exposed to skin-penetrating, larval flatworm parasites of the family Schistosomatidae, that emerge from aquatic snails. Swimmer’s itch is a globally-distributed, allergic condition, of which we know very little regarding local dynamics of transmission. This study aims to gather relevant information on swimmer’s itch in Canada, from multiple perspectives, including the human experience, parasite and host presence and distributions, and insight from historical perspectives.

**Methods:**

Herein we utilize a mixed-methods approach towards examining the environmental health issue of swimmer’s itch in Canadian lakes from a nation-wide viewpoint, with an example from Alberta. We examine the human perspective of having contracted swimmer’s itch through a self-reporting surveillance system implemented over a 5-year period. We also conducted a 3-year species survey of parasites and intermediate snail hosts within lakes in central Alberta and compiled this data with snail and vertebrate (definitive) host survey data from across Alberta to examine potential for future spread. We compare the results from our surveys to a historical review of the literature to examine the extent of swimmer’s itch across Canada and identify where future efforts should be focused.

**Results:**

Over 3800 cases of swimmer’s itch were captured across Canada by the self-reporting surveillance system. Swimmer’s itch cases were reported from every province except Prince Edward Island. Species surveys in Alberta revealed 7 new parasite and host records, with potential for swimmer’s itch to occur throughout most of the province based on host distributions. A review and comparison to the literature has highlighted several knowledge gaps surrounding schistosome species, host species and their distributions and contributions towards swimmer’s itch.

**Conclusions:**

Swimmer’s itch is a greater environmental health hazard across Canada than previous literature would have alluded. This study provides proof-of-concept for the utility of a self-reporting surveillance system for swimmer’s itch in Canada. Recommendations are made towards implementing a systems-thinking approach that incorporates citizen-scientists for future research, management, and policy surrounding swimmer’s itch.

**Electronic supplementary material:**

The online version of this article (10.1186/s12940-018-0417-7) contains supplementary material, which is available to authorized users.

## Background

Cercarial dermatitis, or ‘swimmer’s itch’, is often referred to as an emerging disease, despite the fact there are no data tracking the number of afflicted people [1]. Swimmer’s itch is an allergic rash contracted when swimming in natural water bodies and is by no means a new condition, as historical reports from the literature suggest a global distribution. Nearly 100 years ago, the cause of swimmer’s itch was discovered by Dr. William W. Cort at Douglas Lake, Michigan, when handling snails during collections resulted in the development of a rash. Cort found that it was the larval cercarial stage of avian schistosomes (parasitic flatworms) emerging from their snail host that caused the rash by penetrating the skin, and he described the condition as cercarial dermatitis [2]. Even before Cort’s discovery, cercarial dermatitis was informally described using a variety of names. For example, in 1887, skin conditions resembling cercarial dermatitis were described among Japanese rice farmers, referred to locally as ‘koganbyo’ or ‘lakeside disease’ [3] (other names reviewed in [1, 4]). Swimmer’s itch has been noted to occur in both fresh and saline waters, though it is caused by different schistosome genera cycling through different genera of snail hosts [4].

Although schistosomes in general are noted to cause dermatitis, it is those of the genera *Trichobilharzia* and *Schistosomatium* that are most notably the etiological agents in freshwater ecosystems in North America. *Trichobilharzia* species utilize waterfowl as a definitive host in which they mature into adult worms, while *Schistosomatium* species utilize small, aquatic mammals [4].

A review of the literature exposes the marked historical presence of swimmer’s itch across most of Canada for nearly a century. In Canada, the first report and confirmation of the etiological agent of swimmer’s itch, also commonly referred to as ‘schistosome dermatitis’ in the literature, was by McLeod in 1934 at Clear Lake, Manitoba [5]. Incredibly, this report notes that over 50% of the 55,000 visitors to this lake in the summer of 1933 had contracted swimmer’s itch. McLeod relates this outbreak to the designation of the area of Clear Lake as a National Park that increased visitation dramatically, and therefore, exposure of people to the cercariae, which had likely been there long before [5]. This first report was by far the largest case report found within our literature review, and likely in the history of swimmer’s itch, spurring a series of downstream studies across the nation. Most of these studies focused on identifying the schistosome species and their hosts, sometimes with mention of a swimmer’s itch outbreak, but nearly all reports neglecting to specify how many people were affected and what defined an ‘outbreak’. A timeline of swimmer’s itch across Canada, from the primary literature, shows one or two outbreaks occurs every decade (Additional file 1: Table S1). These reported outbreaks, however, are not from the same province and show no pattern.

The potential for swimmer’s itch to occur as suggested by the presence of schistosome cercariae emerging from snail hosts, or adult worms found in definitive hosts has been indicated for most of Canada [6, 7]. As far back as 1936, W.W. Cort described correspondence with Dr. S.G. Saunders of the University of Saskatchewan, who had reported schistosome positive snails from Paul Lake in British Columbia, the Peace River area of Alberta, Vancouver Island (BC), and Saskatoon, Saskatchewan [8]. Since then, including the outbreaks listed above, all provinces except Prince Edward Island have had either reports of swimmer’s itch or the presence of schistosome parasites published (Additional file 1: Table S1). However, because of the lack of epidemiological data, we have poor estimates of people affected. Beyond the first report of potentially over 25,000 cases, only 280 cases from across all of Canada for the last 64 years can be accounted for. Analogously, these one-time reports of the schistosome species present as the etiological agent for swimmer’s itch in that area leaves a major assumption of a stable and consistent parasite community. Without continuous or even repeated surveillance, we have a poor understanding of these trematode communities and how their populations might fluctuate over time and lead to outbreaks.

Despite the widespread, global distribution of swimmer’s itch, there remains limited understanding of many factors that pertain to the life cycles of schistosome species, their hosts, and what might be limiting their distributions. Canada is greatly endowed with freshwater and hosts a large diversity of waterfowl that utilize wetlands as summer breeding grounds; yet, few studies have examined the parasites of these birds, and direct links to swimmer’s itch outbreaks within the country. In Alberta, previous reports of swimmer’s itch presence have been noted in the literature [8, 9], but details related to schistosome or host species locality, and impact on people has not been well-demonstrated. In fact, only three reports of the presence of schistosomes can be accounted for in Alberta. In 1936, Saunders described to Cort authentic accounts of swimmer’s itch from the Peace River District of Alberta, but there was no mention of schistosome species or hosts [8]. In 1940, Hadwen and Fallis described an outbreak at Elk Island National Park, and investigated potential causes, of which they owed the outbreak to *Cercaria elvae* found emerging from *Lymnaea stagnalis* snails. *Cercaria elvae* was first indicated as the causative agent by Cort, and today it is known as *Trichobilharzia ocellata* [10, 11]. The third account was of the presence of *Dendritobilharzia pulverulenta* from an Eared Grebe prior to 1980, of which no further information of location or possible snail host was mentioned [12]. The story is quite similar for other Canadian provinces, with past work concentrated in Manitoba, and swimmer’s itch reports concentrated around Quebec and in Southern Ontario near the Georgian Bay [6, 8, 13, 14].

To assess whether swimmer’s itch is truly an emerging disease, we need a baseline of information for comparison, especially in consideration of the potential for altered risk in association with climate change. It is predicted that warmer temperatures will affect the occurrence rates of swimmer’s itch, in that warmer temperatures have been associated with increased schistosome developmental rates (more and faster production of cercarial larvae produced in the snail) and therefore, transmission success. Likewise, lake eutrophication can lead to more nutrient availability for snails, enhancing their success and leading to larger populations that can support more schistosome infections (reviewed in [4]). While schistosomes are not the only trematodes infecting snails, and there is still much to be learned about their community ecology, and whether prevalence of snail infection would be greater for schistosomes than other species, increased risk is still a possibility in the future. To address this, we need to better understand the dynamics of species interactions, their relationship to the environment, and the same for human interactions and impact on these natural areas.

We predict that swimmer’s itch is a more widespread problem in Canada than would be revealed by past reports and coverage within the literature. To test this, we gathered swimmer’s itch case reports from individuals through a voluntary, online survey every summer from June 2013 – September 2017. Our aim was to better understand the spread of the issue in Canada, and to determine peaks of occurrence. We also endeavoured to identify Alberta schistosome species and their snail first intermediate hosts to better understand the current and potential future spread of swimmer’s itch through host species distributions in the province.

## Methods

### Web-based self-reporting survey

In 2013, a web-based, self-reporting survey was developed as a surveillance tool to gain an understanding of where and when swimmer’s itch occurred across Canada. The survey was hosted on the website http://swimmersitch.ca/, which also acted as a general information source for people to reference about swimmer’s itch, including the difference between what a swimmer’s itch rash and a cyanobacteria rash look like, the parasite life cycle, and a section for frequently asked questions. The survey was introduced by a brief paragraph to explain the purpose of the survey, followed by an information letter and consent form. The survey entailed 15 questions (Additional file 2: Table S2), asking respondents for information related to the lake they visited and their swimmer’s itch experience. The end of the survey was followed with a general comments section to allow for any other information respondents wanted to provide, including general feelings. The survey also provided a definition of the swimmer’s itch rash:

“An itchy, red, raised rash usually characterized by small reddish pimples that appears after time spent in lakes or ponds. Symptoms can start within a few minutes to 48 hours after being in the water.”

People were not actively recruited to fill out the survey, however, the website and associated survey were advertised through social media (Twitter: @swimmersitch_ca & University of Alberta School of Public Health website: https://www.ualberta.ca/public-health/about/this-is-public-health/this-is-public-health-articles/2014/july/swimmers-itch-in-albertas-lakes), official press releases that resulted in interviews with the Canadian Broadcasting Company (CBC), and other media outlets, postcards pinned to community boards and handed out upon personal communication at lakes, through beach outreach by the Alberta Lake Management Society (ALMS), and through personal communication and presentations at conferences.

In 2014, improvements were made to the survey to gain better information for certain questions. For instance, the question “Did anyone you know also contract swimmer’s itch on the same day?” changed to “How many people in your party also contracted swimmer’s itch on the same day not including yourself?” to change it from a yes/no question to a quantitative response, to better reflect the number of swimmer’s itch cases. We also added the question “To your knowledge, was there a Blue-Green Algae warning at this lake the day you visited?”. The latter question was added to gauge whether their rash might be due to a cyanobacteria bloom as opposed to schistosome cercariae.

### Web survey statistics

Survey responses and other survey statistics were analyzed using R [15], with packages *dplyr* [16], *multcomp* [17], *MASS* [18], and *car* [19]. Because most responses were categorical, we used a Chi-Square Goodness of Fit test to compare the expected to observed values within each question. The expected values were based on the null hypothesis of even distributions of possible responses. To test the difference between those who knew whether swimmer’s itch was a common problem at the lake, stratified by residency status (own property vs. visitor), a Pearson’s chi-squared test with Yates’ continuity correction was used.

We wanted to know if the variable month could be a predictor for when peak transmission of swimmer’s itch occurs. To determine if there was any significant effect of month on the number of cases, a generalized linear model (glm) was used after removing outliers. The outlierTest from the *car* package was used to test for and identify outliers. We used Analysis of Variance (ANOVA) with Likelihood Ratio Test to compare the models, in addition to comparing the Akaike Information Criterion (AIC) and Bayesian Information Criterion (BIC) estimators, and the ratio of residual deviance to degrees of freedom to test for overdispersion. Initial examination of swimmer’s itch case data suggested a negative binomial distribution, as it was highly skewed to the right. However, trying to fit a glm with a negative binomial family resulted in overdispersion (dispersion parameter θ = 3.18 e + 26). The models were then re-fit with the Poisson family and log link to adjust for overdispersion. Once the best model was selected, the *lsmeans* package [20] was used for estimating the least-squared means for the rate of swimmer’s itch, calculate 95% confidence intervals, and to employ a Tukey Contrasts post-hoc test for pairwise multiple comparisons of means among significant predictors. The package *bbmle* [21] was used for extracting model comparison statistics. The package *ggplot2* [22] was used for some graphics.

Several questions allowed for open-ended responses to gain a qualitative understanding of how respondents described the water conditions, the birds they saw in the area, and if they thought information on swimmer’s itch was adequate, and where they retrieved this information. For these questions, all words were gathered into a list, and filtered based on frequency and commonality (e.g. the words and, a lot, about, etc.), as well as relevance to the question. Word lists were uploaded to an online word cloud generator (https://worditout.com/word-cloud/create), filtered, and turned into word clouds that reflected the frequency of word use through word size and color. For the water conditions, criteria were set to a minimum frequency of ten uses within the list, while the question about waterfowl type and information resources were set to a minimum frequency of five.

At the end of the survey was a space for general comments, to allow respondents to voice opinions and add extra information if they wished. The comments were coded qualitatively and grouped into themes and subthemes, for a general idea of both what might be missing from the survey, and to gain insight into perceptions and feelings about swimmer’s itch.

### Map of swimmer’s itch cases

ArcGIS Online and ArcGIS Desktop v. 10.6 ArcMap (Esri, 2017) were used to plot swimmer’s itch reports received across Canada from 2013 to 2017. Swimmer’s itch cases were associated with GPS locations gathered from lake names given in survey reports. These were mapped as point locations atop the World Light Gray Canvas Base [23] and Course_HIST213_North American Rivers/North America (MapServer) layer of North American lakes (http://gis-webfs.bucknell.edu/arcgis/rest/services/Course_HIST213_NorthAmericanRivers/NorthAmerica/MapServer). The Heat Map tool of ArcGIS Online was used to display the relative amount of swimmer’s itch cases at each lake, as individual points were stacked. Thus, the more cases, the redder or whiter the surrounding area was colored as opposed to the blue area with less overlapping points.

### Snail-trematode survey

Snail and schistosome collections were conducted within a larger, field-based survey of snail-trematode associations in central Alberta (2013–2014), as reported in Gordy et al. (2016). This study was extended for a third year (2015), using the same methods. Samples described in this study were derived from all three years of the survey.

### Molecular phylogenetics of schistosome species

Larval schistosome cercariae were identified initially by the presence of eye-spots, upon emerging from their snail intermediate host. Each sample was preserved in either 100% ethanol or 50% RNAlater (Invitrogen) and stored at -20C (ethanol) or 4C (RNAlater). Whole genomic DNA was then extracted from several cercariae of each sample with the DNeasy Blood & Tissue Kit (Qiagen) and used to amplify the Folmer region of the mitochondrial gene, cytochrome *c* oxidase subunit 1 (*cox1*), using primers (dice1F/dice11R and CO1F15/CO1R15) as previously described [24]. Samples were sent to Macrogen Inc. (Korea) for Sanger sequencing in both forward and reverse directions, using the same primers as for initial amplification.

Nucleotide sequences were trimmed for quality using 4Peaks (Nucleobytes), then imported to Geneious R11.1 (https://www.geneious.com/, [25]) for downstream analyses. Forward and reverse sequences were aligned using the Geneious alignment algorithm and default parameters. Consensus sequences were derived from these alignments, and protein translations were checked for stop codons, using the trematode mitochondrial translation code, and to make sure they were all in the same reading frame. The consensus sequences were then identified to genera by using tBLASTn (National Center for Biotechnology Information, U.S. National Library of Medicine) to find sequence matches with the highest percent nucleotide identity. The sequences were then used in multiple sequence alignments with representative schistosome sequences derived from GenBank. Sequences generated from this study have been deposited in GenBank under accession numbers (MH168781-MH168796).

A great deal of previous work has been done on the systematics of the Schistosomatidae [26–29], providing many partial *cox1* sequences in GenBank available for comparison. To avoid problems of substitution saturation within phylogenies, more distantly related genera were analyzed in separate alignments. Ultimately, based on BLAST identities, there were two genera, *Trichobilharzia* and *Schistosomatium*, and these were analyzed separately. Multiple sequence alignments were completed using the Geneious algorithm and default parameters. Ends of the alignment were trimmed to the shortest sequence length. The best nucleotide substitution models for rates of DNA evolution were determined using model testing methods with the software MEGA7 [30] for both alignments. The model GTR + G + I was found to be the best model for phylogenetic analyses for both genera and used as such in downstream Maximum Likelihood (ML) and Bayesian Inference (BI) analyses. Geneious plugins were used for phylogenetic analyses. The following settings were used for BI trees in the MrBayes [31] plugin: chain length = 10,000,000, subsampling frequency = 100,000, heated chains = 4, burn-in length = 1,000,000, heated chain temp = 0.2, priors using unconstrained branch lengths with GammaDir (1, 0.1,1,1). The following settings were used for ML trees in the PHYML [32] plugin: branch support = bootstrap, number of bootstraps = 10,000, transition/transversion ratio = estimated, proportion of invariable sites = estimated, number of substitution rate categories = 4, gamma distribution parameter = estimated, optimized for topology/length/rate, and topology search used = BEST (best of NNI and SPR search). As a confirmation of distance-based methods, and as additional support for clade distinction, the web app Automatic Barcode Gap Discovery (ABGD; http://wwwabi.snv.jussieu.fr/public/abgd/abgdweb.html) was used in combination with a priori assumptions of a 5% cut-off in intraspecific sequence divergence for species delimitation using p-distances calculated in MEGA7. For ABGD, we insert nucleotide alignments and tested all three distance measurements (Jukes-Cantor (JC), Kimura 2.0 (K2), and simple distance) to look for agreements on grouping and prior maximal distance, using a minimum slope of 1.

#### Trichobilharzia

A final alignment of 85 sequences was made using *cox1* genes gathered from 20 sequences from this study and 65 sequences from 17 species of *Trichobilharzia*/Avian Schistosomatids gathered from GenBank. For the phylogenies, *Austrobilharzia variglandis* (AY157196) was used as the outgroup. The final alignment was 432 bp with no gaps.

#### Schistosomatium

A final alignment of 12 *cox1* sequences (9 from this study) was made, with *Schistosoma bovis* (AY157212) as the outgroup. The final alignment was 509 bp and consisted of a few minor gaps due to *S. bovis*.

### Definitive and intermediate host distributions in Alberta

Direct field collections of invertebrates and vertebrate sighting records were derived from the Alberta Biodiversity Monitoring Institute (ABMI) Species and Habitat Raw data depository (http://www.abmi.ca/home/data-analytics/da-top/da-product-overview/Species-Habitat-Data_new.html?scroll=true). The ABMI is a not-for-profit scientific organization that monitors and reports on the status and trend in more than 2500 species and their habitats across the province of Alberta. Data are collected from a systematic grid of 1656 sampling sites spaced 20 km apart across the entire province. Along with data on individual species distributions, detailed sampling protocols for both terrestrial and aquatic ecosystems are publicly available online at www.abmi.ca. Species-level presence (or absence) data collected by ABMI from 2007 to 2015 were used to estimate the distributions of potential snail intermediate and vertebrate definitive hosts across Alberta likely to be associated with schistosome and swimmer’s itch transmission. The species considered as likely to be associated with schistosome life cycles and retained in the final data set were the following: Vertebrates (Waterfowl): *Anas* spp.*: A. acuta, A. americana, A. clypeata, A. crecca, A. cyanoptera, A. discors, A. penelope, A. platyrhynchos, A. streptera; Aythya* spp*.: Ay. affinis, Ay. americana, Ay. collaris, Ay. marila, Ay. valisineria, Mergus merganser,* and *M. serrator;* Vertebrates (Mammals): *Microtus pennsylvanicus,* and *Microtus richardsoni;* Invertebrates (Gastropods): Lymnaeidae: *Lymnaea* spp.*, L. columella, L. stagnalis, L. stagnalis jugularis, Stagnicola* spp.*, S. caperata, S. catascopium catascopium, S. elodes, S. exilis,* Physidae: *Physa* spp.*, P. skinneri,* and *Physella gyrina.* The assessment of these species as potential hosts is based on previous reports in the literature, according to the Host-Parasite Database of the Natural History Museum of London [33]. The GPS coordinates associated with the species within our final dataset were used to map their distributions. We used ArcMap from ArcGIS Desktop v. 10.6 as described above. Within ArcMap on ArcGIS Desktop, we used the Tabulate Intersection tool to get statistics on points that overlapped between vertebrate and invertebrate records as well as swimmer’s itch case records, based on GPS for latitude and longitude with an allowance of 10 km.

## Results

Over the five years this survey has been active (2013–2017), we have received a total of 1316 swimmer’s itch reports from across Canada (*N* = 1302), and a few from the United States (*N* = 14). Including additional cases reported since 2014, this adds to a sum total of 3882 cases of swimmer’s itch captured by the survey.

**Table 1:** Summary of swimmer’s Itch survey reports and cases over five years

Month	May		June		July		August		September				
Year	*Reports*	*Cases*	*Reports*	*Cases*	*Reports*	*Cases*	*Reports*	*Cases*	*Reports*	*Cases*	Total Reports	Additional Cases	Total Cases
2013	NA	NA	19	NA	101	NA	149	NA	25	NA	295	NA	295
2014	0	0	1	2[1]	64	242[13]	75	281[51]	6	14[2]	144	388	532
2015	3	6[2]	78	283[12]	144	540[15]	102	355[17]	18	48[5]	347	892	1239
2016	7	18[6]	49	169[20]	128	442[12]	56	176[11]	6	31[10]	248	596	844
2017	1	3[3]	16	26[7]	152	432[14]	99	185[12]	13	22[4]	280	665	945
Average	2.75	9	32.6	120	117.8	414	96.2	249.25	13.6	28.75	262.8	635.25	771
Difference	6.25		87.4		296.2		153.05		15.15		508.2		

Reports are individual survey events, while cases are the addition of reports and the additional number of people affected with swimmer’s itch at the same time as the person reporting. Brackets after the number of cases represents the maximum number of cases per report at that time

**Fig. 1:**
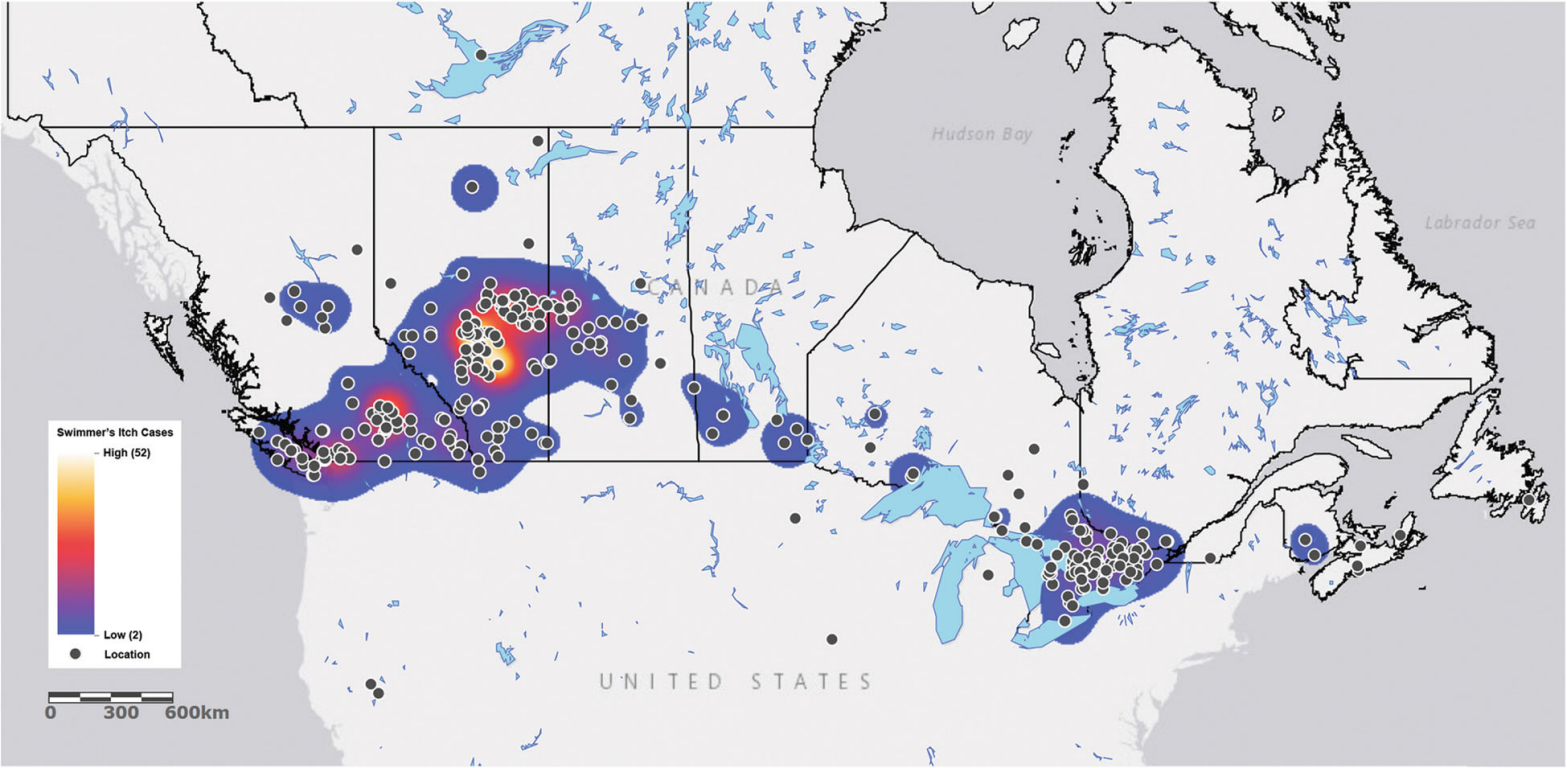
Locations of Swimmer’s Itch Reports Across Canada and the U.S.. Each swimmer’s itch report is represented by a black dot on the map. As many reports are from the same location, the heatmap represents the level of stacking of these reports. Red and white areas represent more reports than blue areas. Single reports have no surrounding colour

We received reports from every province in Canada except Prince Edward Island (Fig. 1). Most reports, however, were from Alberta (610 reports/1935 cases), British Columbia (400 reports/1071 cases), Ontario (159 reports/435 cases), and Saskatchewan (93 reports/311 cases), and were represented every year of the survey. Reports from Manitoba (14 reports/53 cases) occurred every year except 2014. Reports from Quebec (10 reports/ 19 cases) were received in 2013, 2014, and 2016. Reports from New Brunswick (3 reports/13 cases), Nova Scotia (4 reports/7 cases), Newfoundland and Labrador (2 reports/7 cases), and the Northwest Territories (1 report/1 case) occurred less frequently, represented in only one or two years.

**Fig. 2:**
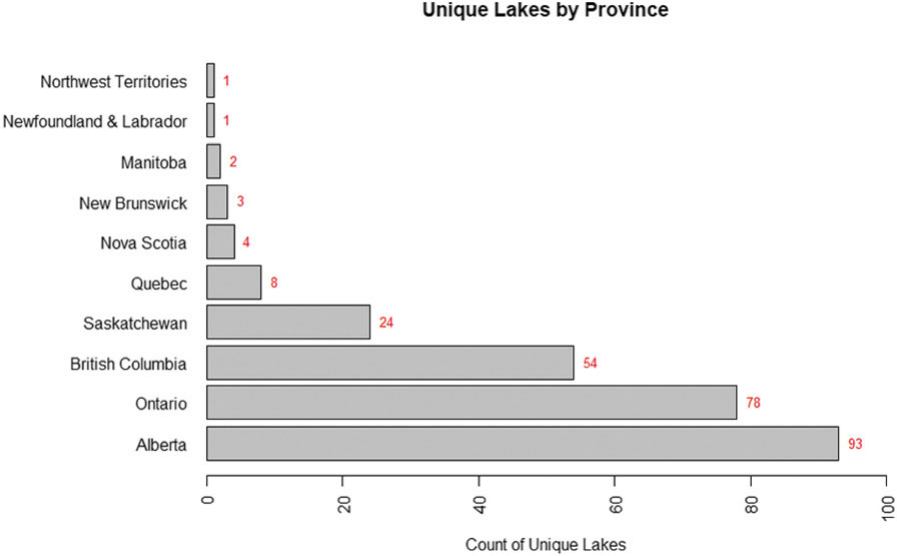
Number of Unique Lakes from which Swimmer’s Itch Reports were Received by Province

Because respondents listed a beach name or a lake name, we did not have enough resolution to provide specific GPS locations for each report. Across Canada, there were a total of 268 unique lakes in which swimmer’s itch was reported to occur (Fig. 2). With the inclusion of several beaches at the same lake, there were 323 unique sites in total.

**Fig. 3:**
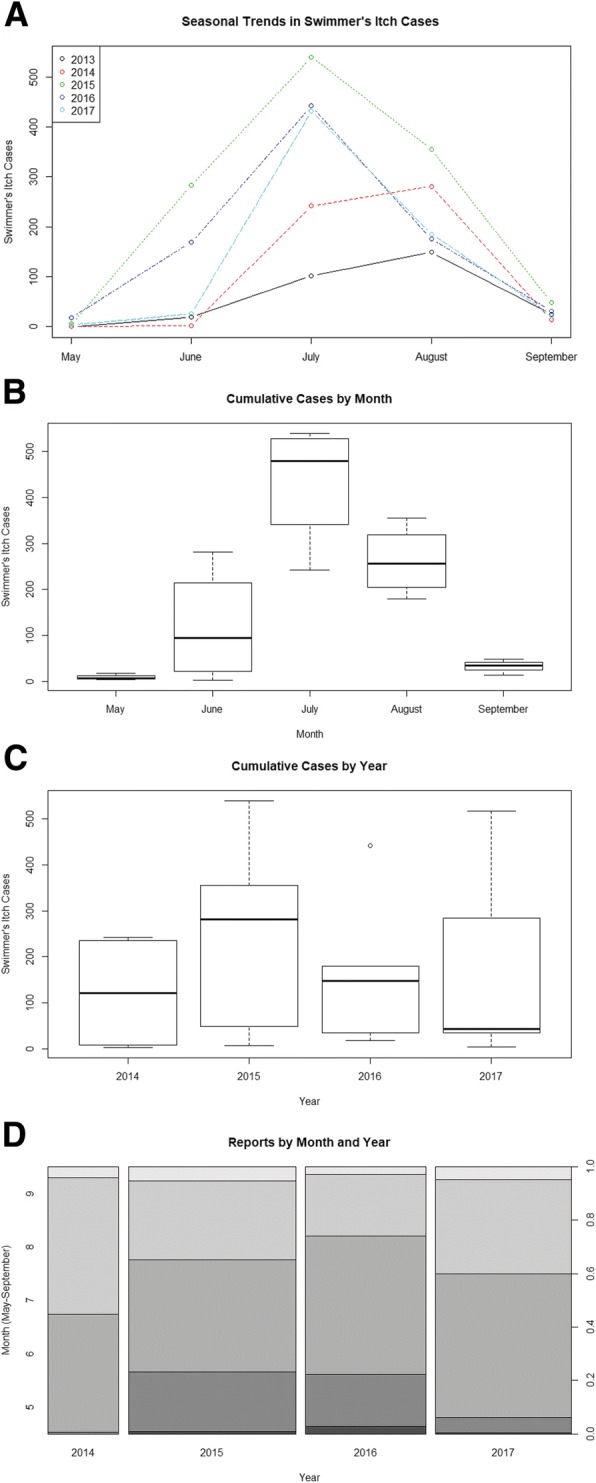
Trends in Swimmer’s Itch Occurrences**. a** The total number of swimmer’s itch cases per month, as gathered by survey reports, are plotted for each year. **b**. Cumulative swimmer’s itch cases by month (2014–2017). **c**. Cumulative swimmer’s itch cases by year; **c**ases for 2013 were not included, as this question was not added to the survey until 2014. **d** Spine plot of swimmer’s itch case proportions by month over four years (2014–2017). Grey-scale partitions within bars are proportions of cases in each month within the year. Width of the bars reflects the total sample size for each year

**Fig. 4:**
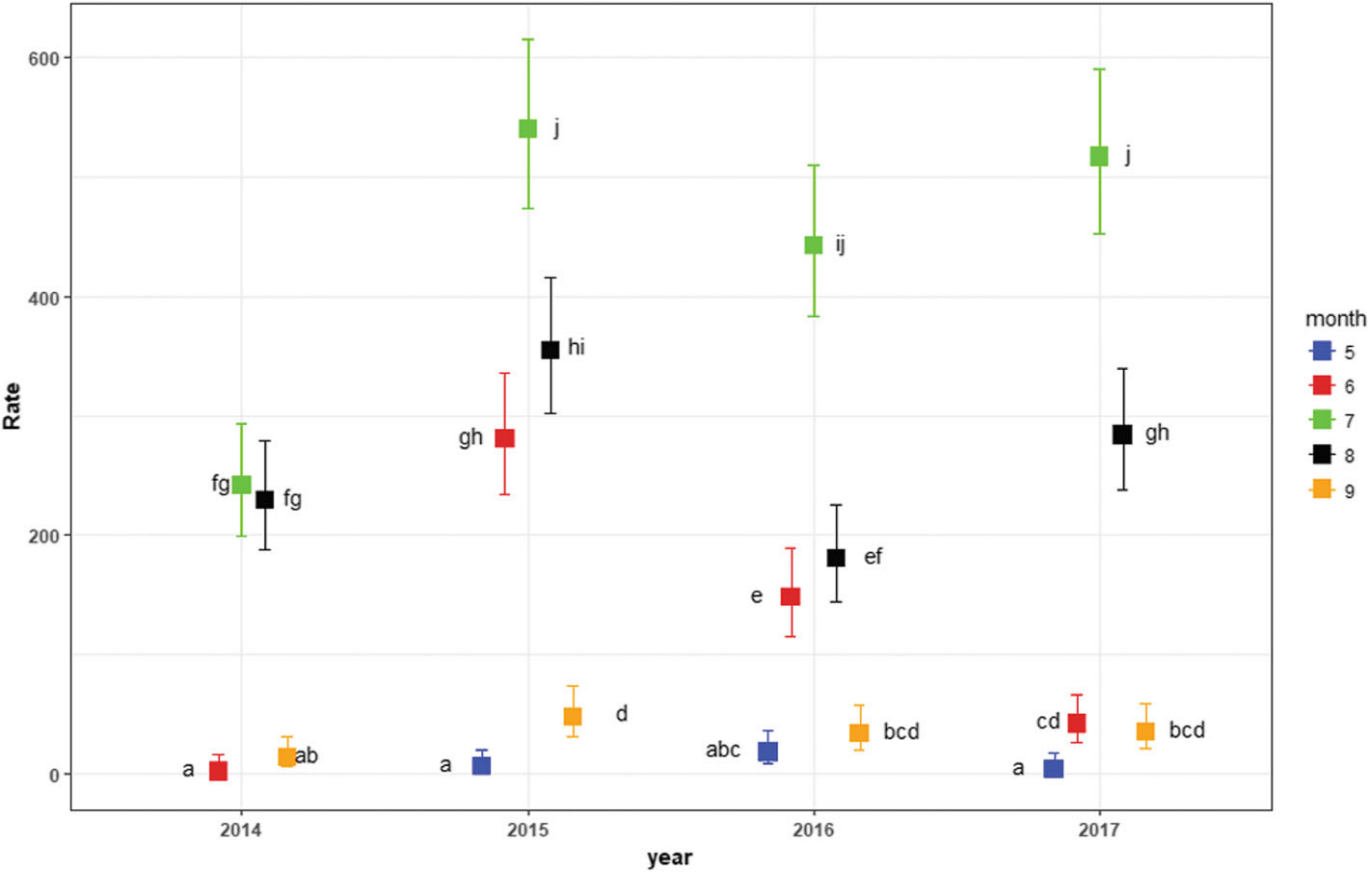
Rates of Swimmer’s Itch Cases Over Time. Least-square means of swimmer’s itch case counts over five months in each year for four years (2014–2017). Boxes indicate the least-square means of the rate. Error bars indicate the 95% confidence intervals around the rate. Rates sharing a letter are not significantly different (Tukey-adjusted comparisons)

We received swimmer’s itch reports from May through September in most years (survey started June 2013) (Table 1). The first two years of the survey trended towards August as the peak of the season for swimmer’s itch, but the last three years moved towards peak occurrences in July (Fig. 3a). The greatest number of occurrences over the course of the survey was 540 cases in July of 2015. This, in combination with cumulative cases by month and by year, suggests that July is an important month for swimmer’s itch occurrence (Fig. 3b), and that 2015 was a particularly big year for survey reports (Fig. 3c and d). However, an important interaction between month and year on the rate of swimmer’s itch cases was revealed from the best fit of the generalized linear models (cases ~ year*month). Simply, the effect of month on the rate of cases depends on the year and vice versa (Additional file 3: Table S3 and Fig. 4). Although July and August both appear to be peak months for swimmer’s itch occurrence, there is more complexity than can be explained by only month for the number of swimmer’s itch cases that may occur, and more importantly, that may be reported.

**Fig. 5:**
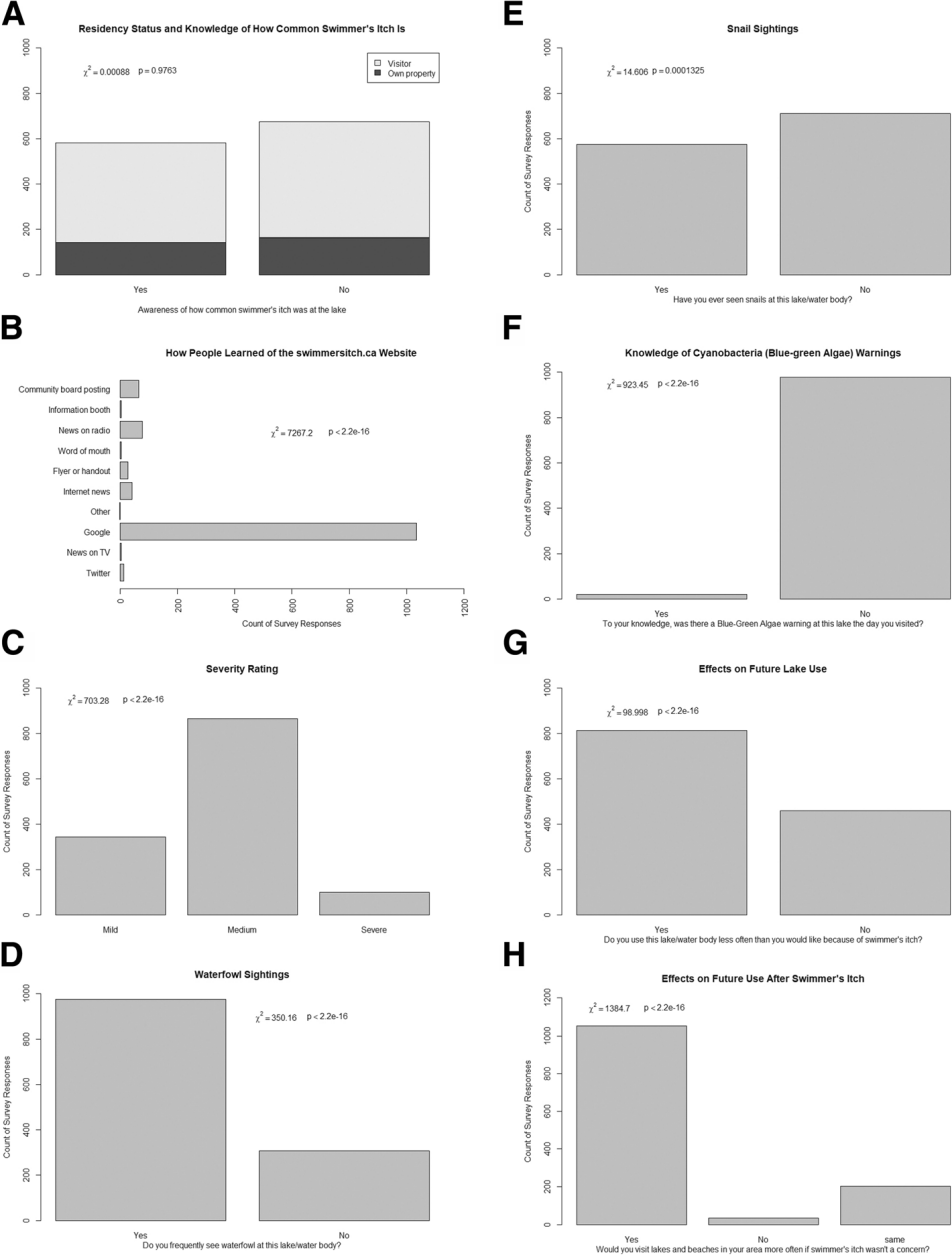
Self-Reporting Survey Results. **a** Residency status and knowledge of how common swimmer’s itch is. **b** How respondents heard about or found the survey. **c** Swimmer’s itch severity rating as identified by respondents. **d** Whether or not respondents sighted waterfowl at the lake during the time they contracted swimmer’s itch. **e** Whether or not respondents sighted snails at the lake. **f** Whether or not respondents were aware of a blue-green algae (cyanobacteria) warning sign at the lake. **g** Potential effects on future use of the lake for recreation after having had swimmer’s itch. **h** Whether respondents would visit more, less, or the same amount if they knew whether or not swimmer’s itch was a risk

**Fig. 6:**
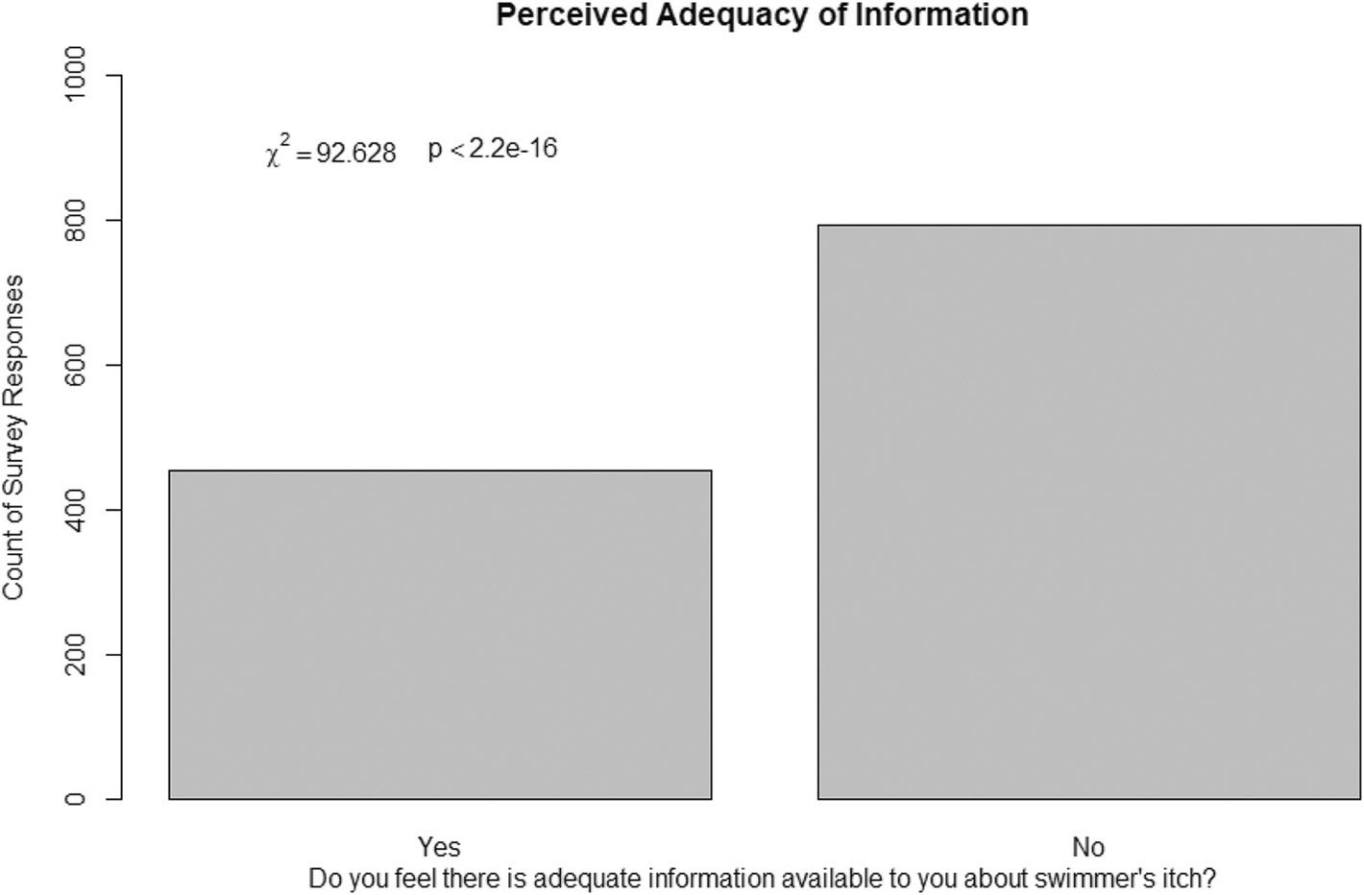
Respondents Opinions on Whether or Not the Amount of Swimmer’s Itch Information Available To Them Was Adequate

**Fig. 7:**
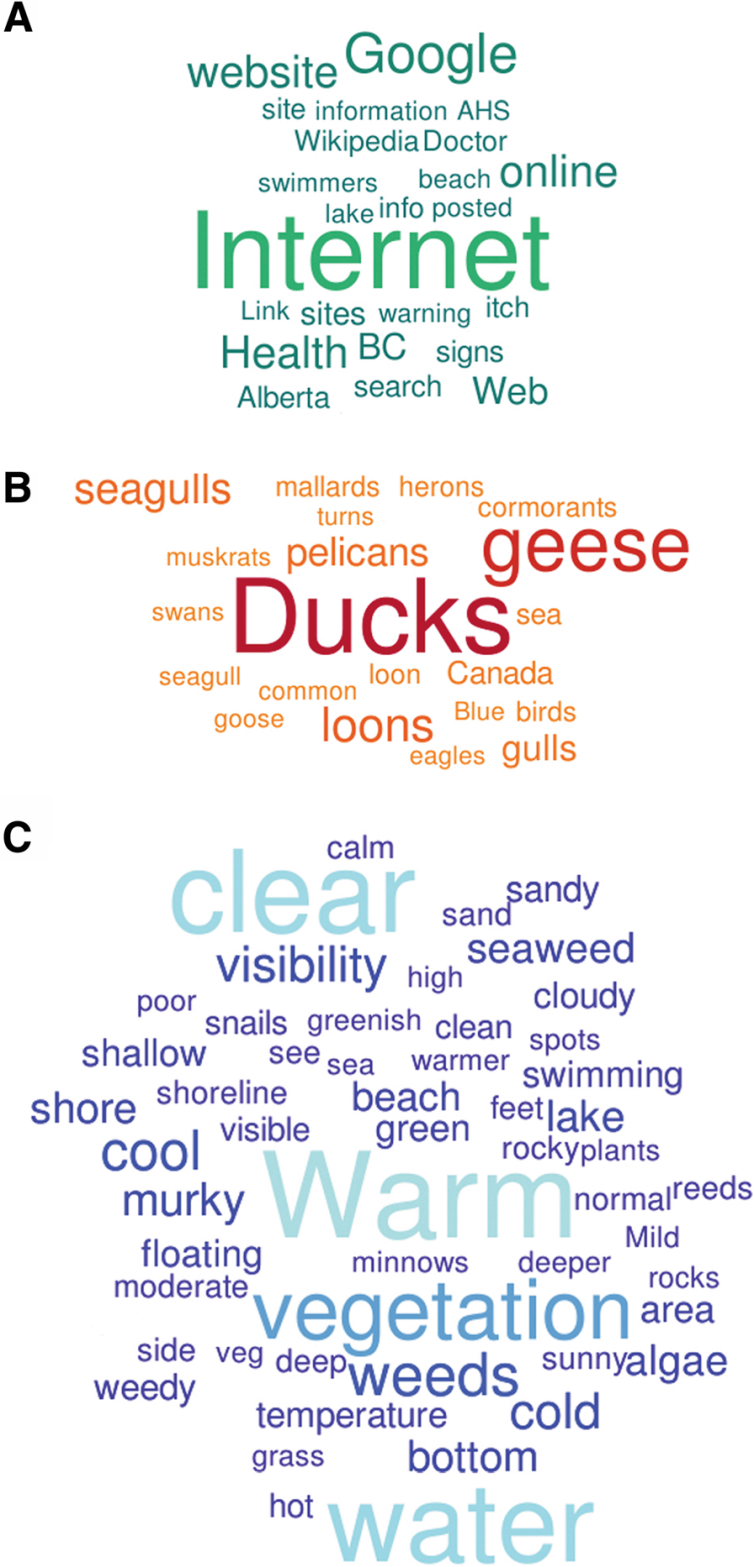
Word Clouds of Most Commonly Used Words in Descriptive Text Answers**. a** Most common sources sought for swimmer’s itch information. **b** Most common waterfowl sightings. **c** Most common descriptors of water quality

Survey respondents were asked a series of questions to help us better understand their general awareness of swimmer’s itch, their perceptions of the environment, and how their experience might affect future use of lakes for recreation. Most respondents were visitors to the lake at which they experienced swimmer’s itch, as opposed to owning property on the lake (Χ^2^(1, *N* = 1315) = 361, *p* < 2.2e-16). Whether or not the respondent owned property at the lake had no significant effect on whether they knew if swimmer’s itch was common at that lake (Χ^2^(1, *N* = 1315) = 0.00088, *p* = 0.9763) (Fig. 5a). Most respondents said they did not feel like the amount of information available to them about swimmer’s itch was adequate (Χ^2^(1, *N* = 1248) = 92.6, *p* < 2.2e-16) (Fig. 6). For those that said there was enough information available, they were asked to provide the resources they commonly consulted in an open-response format. After filtering for frequency and relevance, there were 24 words left, of which the most common was “Internet” (*N* = 109) (Fig. 7a). When referencing the comments, the most common websites used by people were Health Link BC (https://www.healthlinkbc.ca/), Alberta Health Services or other Government of Alberta Websites (https://www.albertahealthservices.ca/), and the website used to host the survey (http://swimmersitch.ca/). When asked how people learned of the survey on our website, nearly all respondents answered “Google” (Χ^2^(9, *N* = 1270) = 7267.2, *p* < 2.2e-16) (Fig. 5b). Google was the most frequently accessed source; however, some people did find out about the survey through advertisement methods such as community board postings, and the news.

When asked to rate their case of swimmer’s itch, most respondents rated their severity as Medium (*N* = 866), defined within the question as having a large rash area, very itchy, and having a burning sensation. Much fewer rated their severity as Mild, or a small rash with some itching (*N* = 342), and even fewer as Severe, and requiring hospitalization or a doctor’s visit (*N* = 100), (Χ^2^(2, *N* = 1308) = 703.28, *p* < 2.2e-16) (Fig. 5c).

A total of 303 respondents provided general comments at the end of the survey. The comments fit into four general codes expressing how the comment section was used: 1. Seeking information about swimmer’s itch, 2. Desires swimmer’s itch warning, 3. Providing more information, and 4. Providing opinion. These codes were further divided into themes and subthemes to represent generalizations of information, and provide a brief overview of respondents’ experiences, opinions, and desires (Table 2). While the counts within each theme are not meant to be truly quantitative, most respondents used the comments section to provide more information (*N* = 232). Nearly half of those were describing their personal awareness of swimmer’s itch, lack thereof, or their personal history of swimmer’s itch. The other half talked more about the details within their case, including the environment, presence or lack of signs, the severity of their itch, and more. Many respondents believed that there should be some sort of warning system in place, but there was little clarity on who they thought should have that responsibility. Prevention methods were mentioned quite often within the themes as well, whether it was their personal method for trying to avoid or treat the rash, or discussion about common prevention methods that don’t work, like showering or towelling off after swimming.

**Table 2:** Thematic summary of survey general comments

Codes and Themes	Code and Theme Counts		Subtheme Counts
Seeking information about swimmer’s itch	11		
Themes			
wants an effective remedy/prevention	5		
other	6		
Desires swimmer’s itch warning	53		
Themes			
generally someone should post warnings	18		
local authorities should post information	14		
signs should be posted at the beach	15		
website should warn people	5		
other	1		
Providing more information	232		
Themes			
clarification on other survey answers	17		
describing personal awareness or lack thereof	42		
Subthemes		unaware previous to report	22
		aware but not expecting it	12
		expects to know through warning system	13
		other	2
describing the situation	163		
Subthemes		description of who got it	63
		severity	57
		location	45
		timing	40
		signs or lack thereof	27
		environment	4
		prevention methods	9
		area of body affected	14
		animals seen	2
		signs or lack thereof	30
		other	90
giving anecdotal advice	16		
Subthemes		swimming location and water quality	8
		methods for prevention and treatment	8
personal history of swimmer’s itch	49		
Subthemes		had it previously	26
		first time with itch at this lake	21
		sensitive to swimmer’s itch	7
		other	2
Providing opinion	66		
Themes			
appreciation for our website	15		
concerned about children	9		
may have to see a doctor	5		
mistrust/fear	6		
wish there were showers/facilities	6		
other	26		

Respondents were asked to describe several variables about the environment in which they were swimming/recreating where they contracted swimmer’s itch, including if they noticed the presence of waterfowl and snails, and what the general water conditions were like. They were also asked if they had been aware of any blue-green algae (cyanobacteria) warnings at the lake at that time. Most respondents reported that they had seen waterfowl at the lake (Χ^2^(1, *N* = 1282) = 350.16, *p* < 2.2e-16) (Fig. 5d). Many also reported the types of waterfowl they had seen, though this was generally “ducks” and “geese”. Among these descriptions, 24 words matched the criteria of having a minimum frequency of 5. By far, the word with the highest frequency was “Ducks” (*N* = 646) (Fig. 7b). This could be because the question was leading, in providing the examples “ducks, geese, etc.”, but it is also likely what most people might be familiar with. In contrast, most respondents reported they had not seen snails. Though the total number of survey responses was quite close to those that had seen snails, their distributions were not even (Χ^2^(1, *N* = 1285) = 14.606, *p* = 0.0001325) (Fig. 5e).

After filtering responses about general water conditions, there were 53 words that met the minimum frequency of ten uses. The word “warm” had the highest frequency (*N* = 438), followed by the words “clear”, “water”, and “vegetation” (Fig. 7c). Respondents were also asked about whether, to their knowledge, there had been a warning about blue-green algae in the area at the time they contracted swimmer’s itch. We wanted to get an idea of the possibility that their rash may have been related to other causes, namely an outbreak of cyanobacteria, which are common in many recreational lakes in Alberta. Nearly all respondents said “No” to this question (Χ^2^(1, *N* = 998) = 923.45, *p* < 2.2e-16) (Fig. 5f).

We wanted to know how swimmer’s itch, and the perception of swimmer’s itch after having experienced it, might affect future lake use and the probability of swimming with and without the known risk. More respondents said they use the lake/waterbody less often than they would like because of swimmer’s itch (Χ^2^(1, *N* = 1273) = 98.998, *p* < 2.2e-16) (Fig. 5g), and nearly all respondents said they would visit beaches and lakes in their area more often if they knew that swimmer’s itch was not a concern (Χ^2^(2, *N* = 1293) = 1384.7, *p* < 2.2e-16) (Fig. 5h). While this result is not very surprising, 205 respondents said that their use would be the same either way, suggesting that swimmer’s itch is not their biggest concern.

Over the three years of the snail collection survey, 35 out of 15,969 (0.219%) snails collected across lakes Wabamun, Buffalo, Isle, and Gull had patent schistosome infections. From 29 of these samples, we were able to collect enough high-quality DNA for sequencing and species identifications. Twenty of 29 samples came back with high nucleotide identities to *Trichobilharzia* species. Both ML and BI trees agreed on major topologies, and placement of these samples within the avian schistosomes. Within ABGD, both JC and K2 distance methods agreed on separation of the alignment into 24 groups (Pmax = 0.0215), while simple distance resulted in 22 groups (Pmax = 0.0215). The two groups that resulted differently from these methods were for *T. stagnicolae* and *T. regenti*. Looking at the pairwise distances, there is not quite enough evidence available to call these different species at this point. For *T. stagnicolae*, there is one sequence that groups separately from the rest (FJ174493). If considered as one group, the average within group divergence is 1.3% with a range of 0–5.5%, which is on the edge of the 5% cut-off in nucleotide divergence for *cox1* we used to delineate species. If the sequence is excluded, the within group divergence is reduced to 0–1.4%. Likewise, for the *T. regenti/*cf. *regenti/haplotype peregra* group, the within group average for the combined set is 1.7% with a range of 0–3.6% nucleotide divergence, which strongly suggests it is one species (Additional file 4: Table S4).

Some other interesting observations highlighted by the phylogenies is that *T. mergi* (JX456171–2) and that identified as *T. sp. var. narochanica* (JQ681538–40), group together and have a within group divergence of 0.4%, and range of 0–0.7% nucleotide divergence, suggesting they are the same species. Also, sequences FJ174485 and FJ174509 group together, though one is labeled as *T. querquedulae* and the other *T. sp. D*, and they are 100% identical across the sequenced region in this alignment. Because these sequences are not grouping closely to the others identified as *T. querquedulae*, this suggests, they are both *Trichobilharzia sp. D*. Sequences for *T. szidati* also grouped as two separate clades, however, if combined, showed 3.0% within group average divergence with a range of 0–4.8% (Additional file 4: Table S4).

**Fig. 8:**
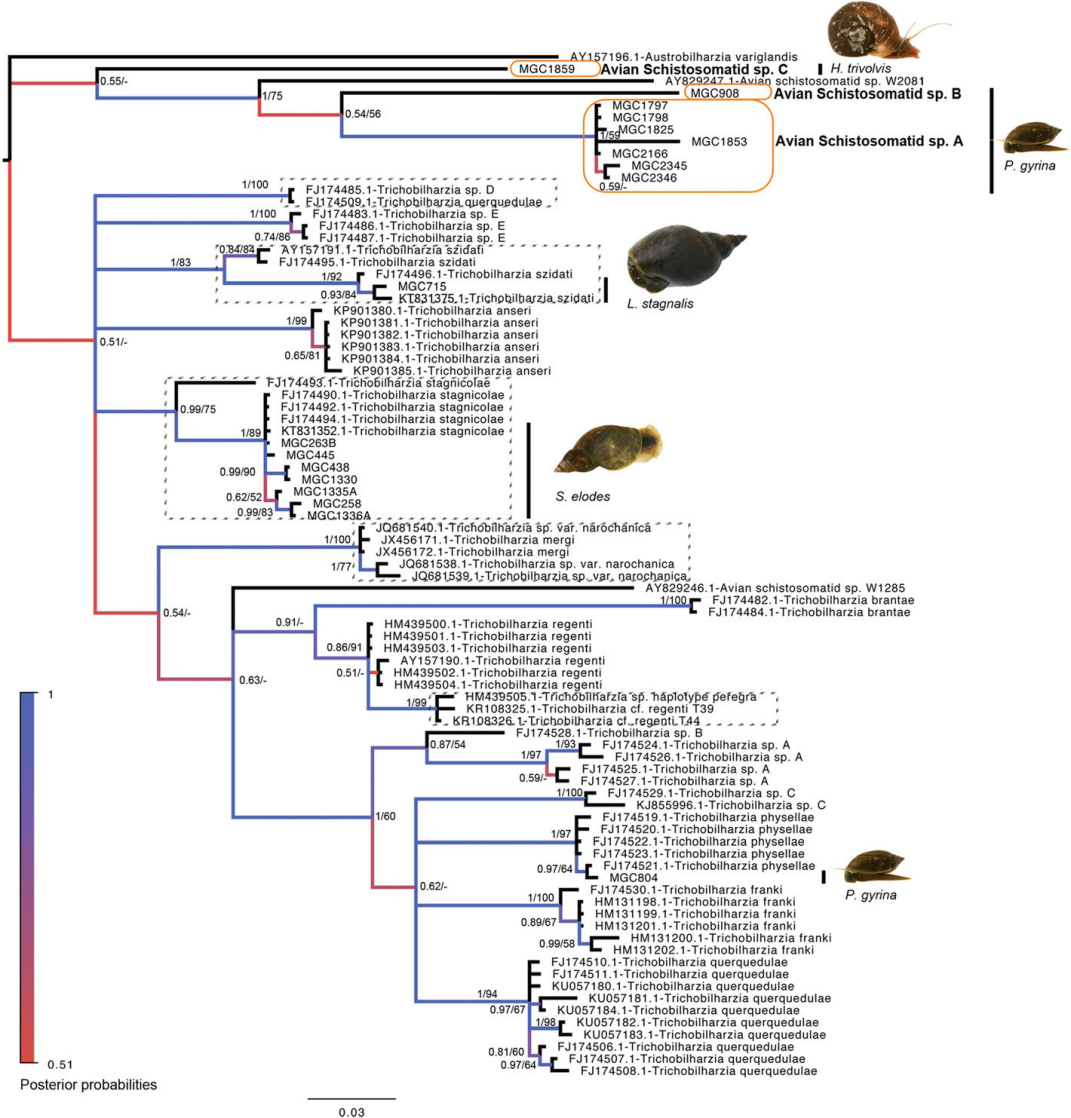
Phylogenetic Tree of Avian Schistosome Genera *Trichobilharzia*. Tree topology is based on Bayesian Inference. Nodal support is indicated with posterior probabilities (shown by coloured branches and associated numeric probabilities) followed by bootstrap support from Maximum Likelihood. GenBank accession numbers precede taxon names. All sequences from this study are labeled with ‘MGC’, and placement is indicated by a black bar to the right of tree. Snail intermediate hosts associated with samples from this study are indicated by a picture to the right

There is strong evidence that the sequences from this study belong clearly to six different species within the avian schistosomes. Three species did not group closely to other *Trichobilharzia* clades, but rather sister to *Avian schistosomatid sp.* W2081 (AY829247) and *Avian schistosomatid sp.* W1285 (AY829246), with average between group nucleotide divergence of 15.2–19.5% between W2081/W1285 and new *Avian schistosomatid spp. A, B,* and *C*. Analyses of these sequences among other avian schistosomatid sequences (*Bilharzia polonica*: AY157186, *Dendritobilharzia pulverulenta*: AY157187, *Gigantobilharzia huronensis*: AY157188, *Ornithobilharzia canaliculate*: AY157194, *Austrobilharzia terrigalensis*: AY157195, *Allobilharzia visceralis*: EF114219, and *Trichobilharzia ocellata:* AY157189) revealed no strong relationship, other than that previously identified within the *Trichobilharzia* tree. All ABGD methods also supported the groups identified in the *Trichobilharzia* tree (Pmax = 0.007743), and p-distances supported separation of species based on a 5% cut-off (*Avian schistosomatid sp. A* within group divergence 0–3.6%; between group divergence for all species 11.5–22.6%) (Additional file 5: Figure. S1). The other species were found to cluster, clearly, within the known groups *T. stagnicolae, T. szidati*, and *T. physellae* (Fig. 8 and Additional file 4: Table S4).

Snail intermediate hosts were as expected for the three identifiable schistosome species: *Lymnaea stagnalis* hosting *T. szidati, Stagnicola elodes* hosting *T. stagnicolae,* and *Physella gyrina* hosting *T. physellae* [4, 26]*.* Of the unknown avian schistosome species (A-C), Physid snails have been noted as a common host for both *Trichobilharzia* and *Gigantobilharzia spp.*; however, *Helisoma trivolvis* snails have only been indicated previously as a possible host for an avian/mammalian schistosome by the identification of emerging cercariae as being brevifurcate-apharyngeate without a finfold [34]. Unfortunately, we do not have photographs of the one cercariae sample that emerged from *H. trivolvis*, identified here as *Avian schistosomatid sp. C*, to look for the presence or absence of a finfold. The purpose of looking for this feature is the distinction between schistosome cercariae and cercariae of spirorchid trematodes that also have eye-spots. Spirorchids are trematodes of turtles, that produce cercariae morphologically similar to schistosomes, and utilize *Helisoma* snails for larval development [35]. Despite the lack of morphological characteristics for our sample, the molecular evidence suggests *Avian schistosomatid sp. C* is not a spirorchid, as nucleotide identity results were highest to *Trichobilharzia spp*., and not *Spirorchis spp.* within GenBank. Even though it is possible this could be an unknown/undescribed species of spirorchid, more molecular similarity to sister species within the same family would be expected. There is strong evidence that this sequence lies within the avian schistosomes, close to *Trichobilharzia spp.*. Further, the only turtle species in Alberta is the Western Painted turtle, that is found in the southernmost part of Alberta, making it unlikely to harbour parasites infecting snails in central Alberta, where the turtles are not present.

**Fig. 9:**
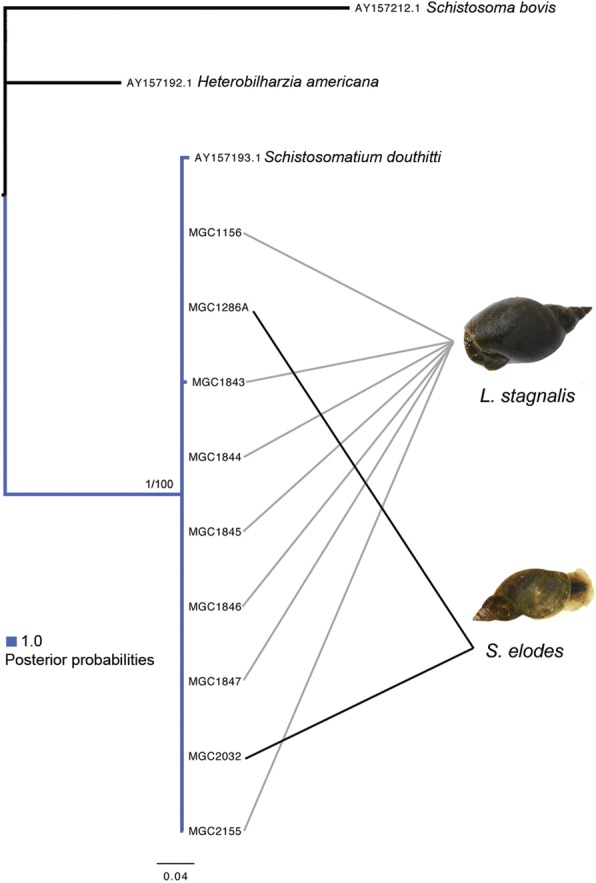
Phylogenetic Tree of Mammalian Schistosomes from Alberta. Tree is based on Bayesian Inference. Nodal support is indicated with posterior probability, by both coloured branches and numbers. GenBank accession numbers precede taxon names. All sequences from this study are labeled with ‘MGC’. Snail intermediate hosts are associated by lines connecting them to specific taxa

High nucleotide identity matches to *Schistosomatium douthitti* were found for nine *cox1* sequences. This identification was confirmed by phylogenetic analysis using an alignment to *S. douthitti* (AY157193), *Heterobilharzia americana* (AY157192) (the other mammalian schistosome species found in North America), and *Schistosoma bovis* (AY157212-out). BI and ML trees agreed on topology. Intraspecific divergence for *S. douthitti* was 0–1.18%, and different from *H. americana* by 22.8–23.27% (Fig. 9). All methods within ABGD agreed on three groups (JC & K2: Pmax = 0.0077, simple: Pmax = 0.0027). Two snail species were found to host these trematodes, *L. stagnalis* and *S. elodes.* A closely related species to *S. elodes, Lymnaea* (*Stagnicola*) *catascopium* and *L. stagnalis* have previously been identified as hosts for *S. douthitti* [36, 37]. Overall, the evidence is strong that these sequences are representing *S. douthitti*.

**Fig. 10:**
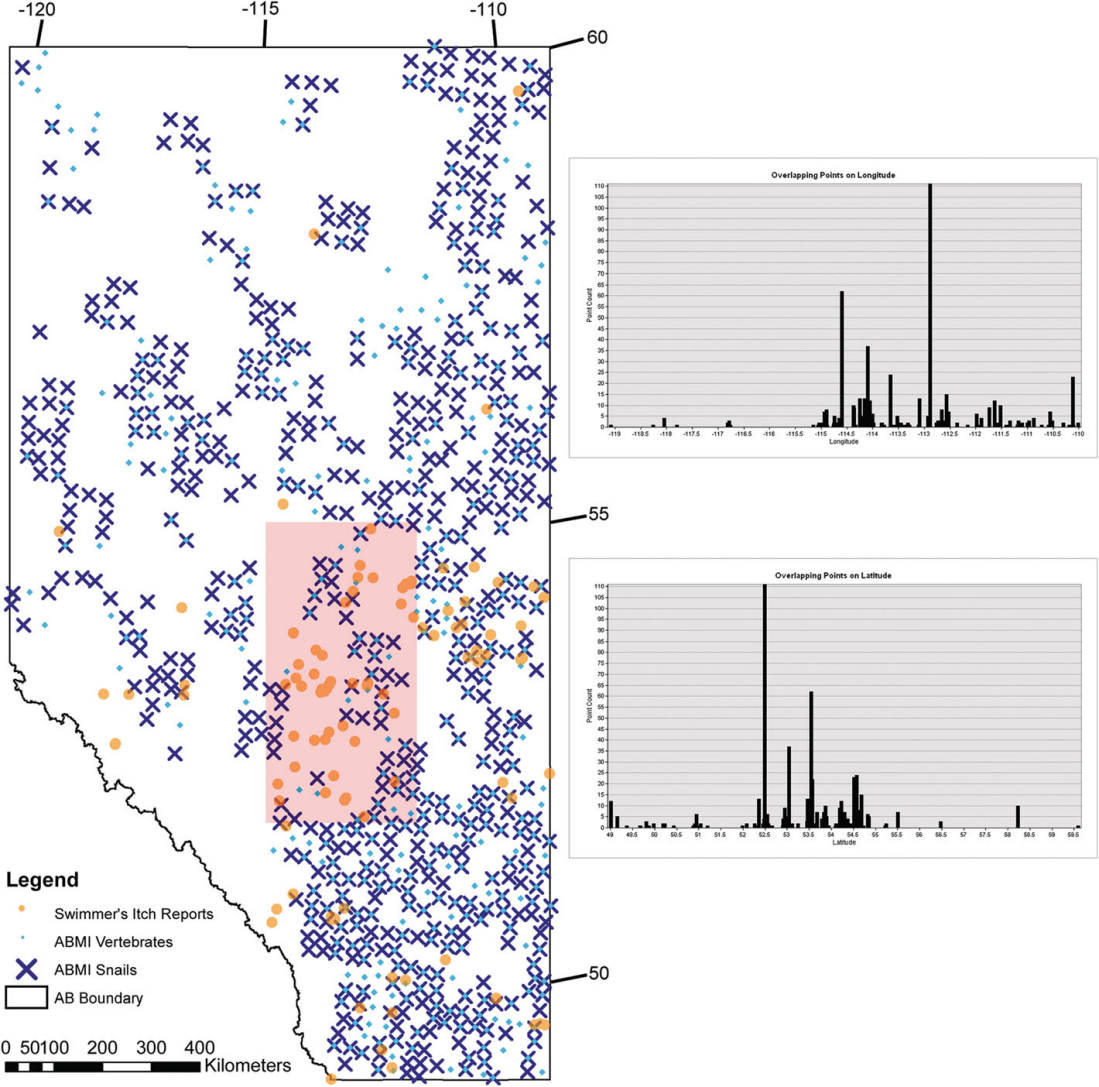
Distribution Map of Our Relative Knowledge of Swimmer’s Itch in Alberta. This map depicts the distributions of where swimmer’s itch cases have occurred (orange circles), and the potential for where swimmer’s itch could occur based on the presence of host species. Vertebrate potential host species are depicted by blue diamonds and invertebrate, gastropod potential host species are depicted by a blue ‘x’. Where the vertebrates and invertebrates overlap is where there is potential for swimmer’s itch transmission. Latitude and Longitude are depicted by tick marks on the outer edge of the map. The graphs on the right-hand side are showing the distribution of overlapping points of latitude and longitude for all three data sets (vertebrate, invertebrate, and swimmer’s itch cases). The x-axis being either points of Latitude or Longitude, and the y-axis describing frequency of overlap, as points are stacked (Min = 1, Max = 111, Sum = 607, Mean = 6.07, SD = 13.3). Most overlapping points, and those with greatest frequency, lie within the red bounded box

The ABMI datasets are quite extensive, and have a broad range across Alberta for available potential host records for both vertebrates and invertebrates. The overlap of vertebrate and invertebrate records also has excellent coverage (Additional file 6: Figure S2, Additional file 7: Figure S3, Additional file 8: Figure.S4, Additional file 9: Figure S5, Additional file 10: Figure S6, Additional file 11: Figure S7, Additional file 12: Figure S8, Additional file 13: Figure S9, Additional file 14: Figure S10). The greatest concentration of swimmer’s itch cases is within central and southern Alberta. Based on Latitude and Longitude values with a 10 km buffer, there were 100 points where all three datasets overlapped. These points were mostly distributed between (52^o^–55^o^) N (Latitude) and - (115^o^–112^o^) W (Longitude) (Fig. 10). This area is the meeting point of three major watersheds in Alberta: The North Saskatchewan, Battle River, and Red Deer River watersheds, where there is a large concentration of lakes, and so it is no surprise that swimmer’s itch is highly concentrated in this area. However, the amount of overlap between vertebrates, invertebrates, and swimmer’s itch cases within this region and other parts of Albert is less than ideal. Many gaps remain, where we have swimmer’s itch occurrences, but no data on hosts, and vice versa. As most of the vertebrate hosts are migratory waterfowl, their potential to move around the province causing their distribution to be relative, and likely broader than what we have data to support at this time. Invertebrates are less geographically mobile than migratory birds, but many of the snail species of concern in Alberta have broad distributions across the Northern hemisphere.

## Discussion

The current study confirms and significantly expands upon our understanding of the distribution of swimmer’s itch across Canada. In fact, many of the lakes at which swimmer’s itch had been reported in past years continue to serve as areas of consistent swimmer’s itch transmission, including Clear Lake (MB), Crescent beach (BC), Cultus Lake (BC), and Lake Nipissing (ON) (Additional file 15: Table S5). However, this study expands upon our past collective distributions within each province to now cover 268 lakes across the country. In Alberta alone, we went from a historical record of one location at Elk Island National Park, to now having case reports from 101 lakes across the province. Considering the size of Canada, the vast number of lakes, and caveats associated with a voluntary survey and localized advertising, it is quite possible we have captured only a small fraction of the actual incidences, albeit far more in a span of 5 years than have been collected over the previous century.

Swimmer’s itch, today, remains a non-reportable condition by national health authorities. The unfortunate result of this status is that we cannot compare our surveillance records to any known incidence rates. Likewise, most recreational areas in Canada do not collect demographic information about the number of people at a lake/area at any one time. The best information we could find was in a report by Statistics Canada, in 2013, of a survey of outdoor activities close to the home, reporting that less than 10% of Canadian households engaged in water-related activities such as “swimming, going to the beach, surfing, scuba diving, [or] snorkeling”. They also found that engaging in these activities was associated with higher socioeconomic status [38]. Unfortunately, this survey does not tell us how many people in Canada live within proximity to water that can be used for recreational purposes, how many people travel for these activities, nor how the data break down regionally. This lack of specific demographic information eliminates the possibility of calculating the true prevalence of swimmer’s itch or back-translating the demographics of our survey responses into representative prevalence. While we suspect that, in our study, localized advertising may have underpinned that the number of reports received from Alberta were greater than from other provinces, the voluntary nature of the survey and fact that it did reach every province makes it difficult to know for sure. This issue highlights an important knowledge gap that should be considered in the future for swimmer’s itch surveillance efforts and policy: we need a better understanding of the true prevalence of swimmer’s itch and how that translates into effects on the recreational choices individuals make and potential downstream effects on cultural values that may have important economic impacts.

One of the greatest questions in regard to swimmer’s itch research is where efforts (and funding) should be focused. It is a condition that does not leave long-lasting, ill-health effects on people, and so Provincial Governments would be less inclined to spend money from their health budget to fund projects that work towards better understanding swimmer’s itch. However, some of the greater effects on people could come from economic impact because of discontinued lake use for recreational purposes or from an unwillingness to utilize outdoor recreational spaces. Furthermore, economic impact might change as a result of climate change, greater anthropogenic impact on natural areas, and eutrophication, which are all potential drivers of increased swimmer’s itch prevalence.

Despite the brevity of health effects from swimmer’s itch, we cannot conclude that there is no impact on the healthcare system. In fact, 100 of our swimmer’s itch respondents had rated their itch as severe and having visited a hospital or doctor. Many had described having to go to their family doctor just to find out what it was. Others had described doctors not knowing what the rash was, despite the history of the patient being in the water. There is potential for swimmer’s itch to have a greater impact on the healthcare system if swimmer’s itch is an emerging disease because there is a general lack of understanding of risk, familiarity with the condition, and knowledge on what to do to prevent or treat it.

While it is not surprising that our coverage of swimmer’s itch cases in Alberta is concentrated where most recreational lakes in the province are located, our analyses would have benefitted from more overlap between the swimmer’s itch cases and other survey records of invertebrate and vertebrate host species in this area. It is apparent that for many lakes in the province, we need more data related to the potential host species that are correlated with swimmer’s itch directly. The same is true for every province in Canada, considering the small amount of information we have related to schistosomes in general.

The current study has now added seven new schistosome and snail intermediate host records to our understanding of the potential etiological agents responsible for swimmer’s itch in Alberta (Figs. 8 and 9, and Additional file 1: Table S1). For a small sampling of lakes within central Alberta, this is substantial diversity for just one family of trematodes. Schistosomes were found at every lake sampled. However, of the total trematode survey completed from 2013 to 2015, schistosomes had a prevalence rate of 0.2%, suggesting that they are likely rare species in comparison to other digenetic trematodes found in Alberta [24, 39]. Alternatively, there could be other factors limiting their ability to infect more snails. This low prevalence rate was surprising to us, especially considering the amount of swimmer’s itch reports we were receiving at the same time from the lakes we were sampling. Other reports from the literature tend to find higher prevalence of schistosomes among snails, although in general less snails are collected and examined, and reports are from a single time point (e.g. 1.24–1.8% in Poland, 3456 and 299 snails examined, respectively [40, 41]; 0.9–1.3% in Argentina, 402 snails examined [28]; 2.6% in Belgium, 270 snails examined [42]). Nevertheless, if the rate of prevalence for schistosomes in snails uncovered through this study is indicative of that for lakes across Alberta, or elsewhere, this holds significance for control measures.

There remain many knowledge gaps across the country on schistosome species presence, hosts and distributions, and life cycle timing. These gaps inhibit our ability to make predictive models, and properly assess risk. It is the basic biology and ecology of these parasites and their hosts that will best inform future risk assessments and potential management strategies. For instance, the discovery that the primary species responsible for swimmer’s itch transmission in Michigan is *T. stagnicolae*, which specifically utilizes mergansers as definitive hosts in this area, has led to control initiatives based on host relocation that has shown successful reduction in snail infection prevalence within control lakes [43]. Whether this type of strategy is possible in Alberta, or elsewhere in Canada, is difficult to say because we have more than one species of schistosome utilizing multiple snail hosts, and many definitive host species within the same lake, adding levels of complexity to the problem. By furthering our understanding of the diversity of host-parasite relationships and ecological drivers behind them, we may be able to develop strategies for control and better surveillance.

In the same light, environmental assessments of lakes in which swimmer’s itch is a common occurrence may prove to provide important links between species presence, life cycle timing, and rates of swimmer’s itch. So far, we know there are higher rates of swimmer’s itch generally in July and August in Canada, but because these are the warmest months, it is also when more people are out on the lakes and swimming. This is not generally helpful information to people trying to decide whether or not to swim.

Considering that only one snail infected with a schistosome is necessary to cause swimmer’s itch, the concept of control or management of the issue is an arduous task. Past efforts to kill off the snail population, or even treat the birds with anthelminthics have been costly, labor-intensive, and unsuccessful in the long-term [44, 45]. These failures necessitate new, innovative solutions.

There are several areas in need of further research and development. First is the need for an accurate and sensitive method to monitor for the presence of schistosome cercariae in the water, as snail collections have proven to be an inefficient and ineffective method for surveillance. Work has already begun to develop and test methods using qPCR-based strategies [43, 46]. Molecular detection of larval cercariae in the water may overall be a better assessment of risk, and this strategy would allow for timely communication of risk to lake users and is able to capture all schistosome species within a sample regardless of which species they are.

Second, and perhaps the most important, is the need for better communication and education for lake users, lake managers, and health authorities on swimmer’s itch as a risk in natural water bodies. While many recreational lakes do use signs near public beaches to warn about swimmer’s itch, this is not a standard. Often, these signs will also do a poor job at describing the situation or what a person should be expecting (personal experience). As most people using the lakes for recreation are visitors, there should be no expectation that they should know the history of swimmer’s itch at a given site, especially considering that even from published studies, we were unable to determine whether it is a risk at a particular lake. Many survey respondents, after having experienced swimmer’s itch, noted that they believed there should have been a warning sign. The development of an effective warning sign is a tricky business. The human information processing model describes multiple steps a person must go through to change their behaviour: first, the warnings must capture the person’s attention, then they must comprehend the information, then believe the information and not dismiss it based on beliefs or attitudes, and finally, the person must be motivated to comply [47]. While the first and last steps are easy to accomplish with a health-related sign, the middle two steps are quite challenging in this context. For one, many people believe that swimmer’s itch is caused by algae (personal communication), which is not surprising, as often algae or cyanobacterial blooms are quite obvious, visible, and often have a negative stigma attached. Describing that swimmer’s itch is caused by a microscopic larval parasite with a complex life cycle that involves both snails and birds/mammals is challenging. The explanation requires adequate comprehension of what a parasite is, what a larval cercaria is, and how a complex life cycle works. Because it cannot be seen without the aid of a microscope, schistosome cercariae as the cause of swimmer’s itch is less believable and more abstract than something like algae that a person can see in the water. It is possible that the use of poorly developed swimmer’s itch signs might act as a deterrent to recreational use of lakes, which, from an economic perspective, is not ideal. Therefore, it is of utmost importance that if swimmer’s itch warning signs are used at lakes, that they are accompanied with educational campaigns and that the information is made accessible. Other challenges arise in defining swimmer’s itch from a clinical perspective (how many papules does a person need to have?), defining an outbreak (how many people must contract it?), and ensuring communication between lake users and health authorities.

## Conclusions

From the success of our voluntary survey, it is apparent that people are willing to participate in efforts to address the swimmer’s itch problem. There are many avenues for future swimmer’s itch research that could benefit from a citizen science approach, considering that surveillance across the entire country is needed. We greatly encourage the development of applications that help towards identifying waterfowl and aquatic snails, as this could provide a strong foundation from which to develop more specific strategies for research and management options. Including people in the research on topics that directly affect them can also be a good strategy for education and communication. A national web-based surveillance system could help us better track swimmer’s itch, organize the information, and help us understand where and when it is occurring in a real-time format. This study strongly supports the concept that a citizen science approach can be an effective strategy towards the continued surveillance of swimmer’s itch in Canada. Ultimately, this problem would lend well to a systems-thinking approach in that it is too complex for an individual group to conquer. This problem requires a transdisciplinary, collaborative approach that incorporates the public into surveillance programs for improved overall management and communication.

## Additional files

Additional file 1: **Table S1.** Literature Review of Schistosomes, Hosts, and Locality within Canada, and Historical Outbreaks of Swimmer’s Itch. (PDF 613 kb)

Additional file 2: **Table S2.** Swimmer’s Itch Survey Questions. (PDF 388 kb)

Additional file 3: **Table S3.** GLM Model Comparisons for Effects of Year and Month on Swimmer’s Itch Occurrences. The metrics within this table were used to compare deviance across models and to decide the best fit model. Abbreviations: Df/df = Degrees of Freedom, Resid. = residual, Dev. = Deviance, loglik = log likelihood, AIC = Akaike Information Criterion, BIC = Bayesian Information Criterion, dAIC = delta AIC. The best model was selected on the lowest AIC and BIC, having a residual deviance equal to the residual degrees of freedom, and a high log likelihood. The best model was therefore Model 1. (PDF 323 kb)

Additional file 4: **Table S4.** Estimates of Evolutionary Distance Between Pairs of Groups of *Trichobilharzia* and other Avian Schistosomatid Species. The number of base differences per site from averaging over all sequence pairs between groups are shown. Standard error estimate(s) are shown above the diagonal. Within group divergence is shown on the diagonal and in bold. The range of within group divergence is in parentheses after the group name on the first column. The rate variation among sites was modeled with a gamma distribution (shape parameter = 1). The analysis involved 85 nucleotide sequences. Codon positions included were 1st + 2nd + 3rd + Noncoding. All positions containing gaps and missing data were eliminated. There were a total of 417 positions in the final dataset. Evolutionary analyses were conducted in MEGA7 [30]. (PDF 459 kb)

Additional file 5: **Figure S1.** Phylogenetic Tree of Avian Schistosomes. Tree topology is based on Bayesian Inference. Nodal support is indicated with posterior probabilities (shown by coloured branches and associated numeric probabilities) followed by bootstrap support from Maximum Likelihood. GenBank accession numbers precede taxon names. All sequences from this study are labeled with ‘MGC’. (PDF 15551 kb)

Additional file 6: **Figure S2.** *Anas spp.* Distributions Across Alberta Wetlands. Distributions of 9 *Anas spp.* (waterfowl/ducks) as collected by ABMI. (PDF 4716 kb)

Additional file 7: **Figure S3.** Combined Distributions of *Anas spp.* Across Alberta Wetlands. (PDF 3152 kb)

Additional file 8: **Figure S4.** *Aythya spp.* Distributions Across Alberta Wetlands. Distributions of 5 *Aythya spp.* (waterfowl/ducks) as collected by ABMI, both individual and in combination. (PDF 4377 kb)

Additional file 9: **Figure S5.** *Merganser spp.* Distributions Across Alberta Wetlands**.** Distributions of 2 species of diving ducks as collected by ABMI. Both individual and combined distributions are reported. (PDF 3281 kb)

Additional file 10: **Figure S6.** *Microtus spp.* Distributions Across Alberta Wetlands**.** Distributions of 3 species of Muskrat as collected by ABMI. Both individual and combined distributions are reported. (PDF 3594 kb)

Additional file 11: **Figure S7.** Lymnaeid Snail Distributions Across Alberta Wetlands. (PDF 3580 kb)

Additional file 12: **Figure S8.** Combined Lymnaeid Snail Distributions Across Alberta Wetlands. (PDF 2768 kb)

Additional file 13: **Figure S9.** Physid Snail Distributions Across Alberta Wetlands. (PDF 2720 kb)

Additional file 14 **Figure S10.** Planorbidae Snail Distributions Across Alberta Wetlands. (PDF 3638 kb)

Additional file 15: **Table S5.** Lake Locations of Swimmer’s Itch Reports. (PDF 368 kb)

## Abbreviations

ABGD: Automatic Barcode Gap Discovery
ABMI: Alberta Biomonitoring Institute
AIC: Akaike Information Criterion
ALMS: Alberta Lake Management Society
ANOVA: Analysis of Variance
BI: Bayesian Inference
BIC: Bayesian Information Criterion
CBC: Canadian Broadcasting Company
glm: generalized linear model
JC: Jukes-Cantor
K2: Kimura 2
ML: Maximum Likelihood
spp.: Species
