# Supplementary material for: Swimmer’s itch in Canada: a look at the past and a survey of the present to plan for the future
Source: Environ Health. 2018 Oct 25;17:73. doi: 10.1186/s12940-018-0417-7 (PMC6203143; doi:10.1186/s12940-018-0417-7)
Supplement: Supplementary file 1 — Table S1. Literature Review of Schistosomes, Hosts, and Locality within Canada, and Historical Outbreaks of Swimmer’s Itch. (PDF 613 kb) [file 12940_2018_417_MOESM1_ESM.pdf]

Province	Schistosome spp.	Intermediate Hosts	Definitive Hosts	Location/Outbreaks/Cases	Reference(s)
Alberta				Authentic records of dermatitis at Peace River district[§]	Cort, W.W. (1936) Am. J. Hyg. 24(2):318-333
	Cercaria elvae[†]	*Lymnaea stagnalis*		Summer 1939: Outbreaks in Lakes at Elk Island Park	Hadwen, S. & Fallis, A.M. (1940) Can. Pub. Health Assoc. 31(1):30
	Dendritobilharzia pulverulenta		Eared Grebe (*Podiceps nigricollis*)		Vande Vusse, F.J. (1980) J. Parasitol. 66(5):814-822
	Trichobilharzia stagnicolae	*Stagnicola elodes*		Summer 2013-2014: Isle Lake	This study
	Trichobilharzia szidati	*Lymnaea stagnalis*		Summer 2013-2014: Gull Lake and Buffalo Lake (The Narrows)	
	Trichobilharzia physellae	*Physella gyrina*		August 2013: Lac la Nonne	
	Avian Schistosomatid sp. A	*Physella gyrina*		Summer 2015: Isle Lake and Buffalo Lake	
	Avian Schistosomatid sp. B	*Physella gyrina*		August 2013: Lac la Nonne	
	Avian Schistosomatid sp. C	*Helisoma trivolvis*		July 2015: Wabamun Lake	
	Schistosomatium douthitti	*Lymnaea stagnalis & Stagnicola elodes*		Summer 2013-2015: Gull Lake, Wabamun Lake, Buffalo Lake (The Narrows)	
British Columbia	*Cercaria elvae*[†]	*Lymnaea stagnalis wasatchensis*		Paul Lake	Cort, W.W. (1936) Am. J. Hyg. 24(2):318-333
	Trichobilharzia adamsi	*Physa cf. coniformis*	Peking ducklings*	Summer 1950: Severe outbreak at Cultus Lake	Edwards & Jansch (1955) Can. J. Zool. 33:182-194
	Cercaria columbiensis	*Physa cf. coniformis*			
	Cercaria stagnicolae[‡]	*Lymnaea emarginata angulata*		Summer 1963: Two outbreaks at Cultus Lake	Howard, T.E. & Walden, C.C. (1965) J. Appl. Ecol. 2(1):121-135
	Cercaria physellae[¶]	*Physa spp. (P. ampullacea, P.coniformis, P.occidentalis)*			
	Trichobilharzia stagnicolae	*Stagnicola catascopium*	Common Merganser (*Mergus merganser*)	Cultus Lake	Leighton, B.J., et al. (2000) Parasitol. Int. 49(1):9-17
	Trichobilharzia physellae	*Physa sp.*	Common Merganser (*Mergus merganser*)	Cultus Lake	
	Gigantobilharzia sp.	*Gyraulus parvus*	Unidentified Blackbird	Cultus Lake	
	Austrobilharzia variglandis	*Ilyanassa obsoleta*		Summer 2001: 36 cases; Summer 2002: 44 cases at Crescent Beach	Leighton, B.J. et al. (2004) Env. Health Rev. 48:5-13
Saskatchewan	*Cercaria elvae*[†]	*Lymnaea stagnalis jugularis, L. pallustris nuttaliana, & Physa sp.*		Found widely across the province[§]	Cort, W.W. (1936) Am. J. Hyg. 24(2):318-333
Manitoba	*Cercaria elvae*[†]	*Lymanea stagnalis jugularis*		Clear Lake	Swales, W. (1936) Can. J. Res. 14d(1):6-10
	Cercaria sp.	*Stagnicola emarginata canadensis*			
	Cercaria wardlei	*Limnaea obrussa & Stagnicola emarginata canadensis*		Summer 1933: Over 55,000 visitors to this lake, of which over 50% contracted swimmer's itch at Clear Lake	McLeod, J.A. (1934) Can. J. Res. 10(4):394-403; McLeod, J.A. (1940) Can. J. Res. 18d(1):1-28
	Cercaria stagnicolae[‡]	*Stagnicola emarginata canadensis*			McLeod, J.A. (1940) Can. J. Res. 18d(1):1-28
	Cercaria dermolestes sp. nov.	*Stagnicola palustris elodes*		Southern Manitoba	
	Ornitohobilharzia aviani sp. nov.		Ring-billed Gulls (*Larus delawarensis*) & Herring Gulls (*Larus argentatus*)	Clear Lake, Lake Winnipeg, Lake Winnipegosis	
	Ornitohobilharzia filamenta sp. nov.		Ring-billed Gulls (*Larus delawarensis*) & Herring Gulls (*Larus argentatus*)		
	Pseudobilharzia querquedulae[¶]		Blue-winged Teal (*Anas discors*)		
	Microbilharzia mantobensis[‖]		Canvas back duck (*Aythya valisineria*)	Lake Frances	
	Microbilharzia canadensis[‖]		Canvas back duck (*Aythya valisineria*)		
	Microbilharzia lari		Ring-billed Gulls (*Larus delawarensis*) & Herring Gulls (*Larus argentatus*)	Lake Winnipeg & Clear Lake	
	Cercaria elvae[†]	*Lymnaea stagnalis*		Summer 1961: Severe outbreaks at several uncontrolled lakes; Schistosomes found in Lake Norris, Lake Winnipeg, and Lake Manitoba	Farley, J. (1962) Can. J. Zool. 40:131-133
	Schistosomatium douthitti	*Lymnaea stagnalis*	Muskrat *(Microtus pennsylvanicus)*	Southern Manitoba	
	Gigantobilharzia lawayi		Ring-billed Gulls (*Larus delawarensis*) & Herring Gulls (*Larus argentatus*)	Twin beaches at Lake Manitoba, Grand beach at Lake Winnipeg, Red River at St. Andrews, and Moose Lake	Farley, J. (1964) Thesis: University of Manitoba

	Gigantobilharzia gyrauli	Physa gyrina	Red-winged Blackbird (*Agelaius phoeniceus*), Yellow-headed Blackbird (*Xanthocephalus xanthocephalus*), Brown-headed Cowbird (*Molothrus ater*), Common Grackle (*Quiscalus quiscula*)	Long Lake, Meadows, St. Andrews, Norris Lake, North Shoal Lake, Twin beaches at Lake Manitoba	
	Gigantobilharzia totani		Greater Yellow-legs (*Totanus melanoleucus*), Lesser Yellow-legs (*T. flavipes*)	Lake Manitoba, Shoal Lake, Meadows, Moose Lake	
	Trichobilharzia querquedulae		Blue-winged Teal (*Anas discors*), Shoveller (*Spatula clypeata*)	Southern Manitoba	
	Austrobilharzia lari		Bonaparte's Gull (*Larus philadelphia*)	Southern Manitoba	
	Ornithobilharzia canaliculata		Bonaparte's Gull (*Larus philadelphia*)	Southern Manitoba	
				Summer 1977: 6 cases investigated from Clearwater Lake and St. Malo, with additoinal outbreak reports from Gull Lake and Hecla Island	Sekla, L. et al. (1978) Can. J. Pub. Health 69(6):475-480
	Trichobilharzia brantae	*Gyraulus parvus*	Snow Goose (*Chen caerulescens*)	Churchill	Brant, S.V. & Loker, E.S. (2009) J. Parasitol. 95(4):941-963
	Trichibilharzia sp. D	*Stagnicola sp.*			
	Trichobilharzia sp. E	*Stagnicola sp.*	Northern Pintail (*Anas acuta*)		
<u>Ontario</u>				Summer 1954: 53 cases at Lake Nipissing	Mitchell, J.C. (1954) A.M.A. Archives of Dermatology and Syphilology 70(6):805-808
	Schistosomatium douthitti	*Lymnaea stagnalis apressa & Lymnaea palustris elodes*		Chaffey's Locks, Crosby, Cameron Lake, Glen Arm, Reaboro, Newcastle, Rondeau Park, Peterboro, Black Lake	Bourns, T.K.R. (1961) Can. J. Zool. 39(1):43-46
<u>Quebec</u>	*Trichobilharzia cameroni sp. nov.*	*Physa gyrina*	Canaries*, Ducklings*, Pigeons*	Summer 1949: Outbreak near Montreal in vicinity of Ste. Anne de Bellevue and along the Ottawa River	Wu, L. (1953) Can. J. Zool. 31:351-373
				Summer 1988: 74 cases from 5 regions - Soit Hull, Portneuf, Lac St-Francois, Rimouski, and Rouyn-Noranda	Levesque, B. (1990) Can. J. Pub. Health 81(4):329-330
	Cercaria ocellata [†]	*Physa gyrina*		Summer 1998: Outbreak at Lac Nairn; Summer 1999: 63 cases from Lac Beauport	Levesque, B. et al. (2002) Epi. Infect. 129(2):379-386
<u>New Brunswick</u>	*Trichobilharzia stagnicolae*	*Lymnaea emarginata*		Lake Magaguadavic & Lake Utopia	Farley, J. (1967) Can J. Zool. 45(6):1300-1302
	Cercaria catascopii n. sp.	*Physa gyrina*		Prior to 1976: 3 cases at Lake Magaguadavic, 2 cases at Lake Utopia, & 1 case at Lake Chamcook; all lakes had snails with identified schistosome species	Scott, M.E. & Burt, M.D. (1976) Can. J. Zool. 54:2200-2207
<u>Nova Scotia</u>	*Trichobilharzia stagnicolae*	*Lymnaea emarginata*		Lake Mush-a-Mush & Lake	Farley, J. (1967) Can J. Zool. 45(6):1300-1302
<u>Prince Edward Island</u>[δ]	*Austrobilharzia variglandis*	*Nassarius obsoletus*		Bay of Fundy	Farley, J. (1967) Can J. Zool. 45(6):1300-1302

‡Now *Trichobilharzia stagnicolae*
† Now *Trichobilharzia ocellata*
†Now *Trichobilharzia physellae*
§ Now *Austrobilharzia variglandis* or *A. terrigalensis* according to Farley, J. (1971) J. Helminthol. XLV:289-320
δNoted as derived from personal communication
*Experimental infections
