# Supplementary material for: Swimmer’s itch in Canada: a look at the past and a survey of the present to plan for the future
Source: Environ Health. 2018 Oct 25;17:73. doi: 10.1186/s12940-018-0417-7 (PMC6203143; doi:10.1186/s12940-018-0417-7)
Supplement: Supplementary file 2 — Table S2. Swimmer’s Itch Survey Questions. (PDF 388 kb) [file 12940_2018_417_MOESM2_ESM.pdf]

Question	Response Types or Options
Name of lake/water body	Open Response
Where is the lake/ water body located (city, province, country)?	Open Response
Date you contracted swimmer's itch	Open Response
How many people in your party also contracted swimmer's itch on the same day not including yourself? §	Open Response
How would rate the severity of this case of swimmer's itch?	Mild (small rash, some itching)/ Medium (large rash area, very itchy, burning sensation)/Severe (went to doctor/hospital it was so bad)
To your knowledge, is swimmer's itch a common occurrence at this lake/water body?	Yes/No
Do you own property at this lake/water body, or do you visit?	Own property/Visitor
Do you frequently see waterfowl at this lake/water body?	Yes/No
If yes, please list the types if you know them (ducks, geese, etc.)	Open Response
Have you ever seen snails at this lake/water body?	Yes /No
Describe the water conditions (Temperature, visibility, amount of vegetation, etc.)	Yes/No
To your knowledge, was there a Blue-Green Algae warning at this lake the day you visited?	Yes/No
Do you use this lake/water body less often than you would like because of swimmer's itch	Yes/No
Do you feel there is adequate information available to you about swimmer's itch?	Yes/No/Do not think information is necessary
If you answered yes, which resources do you consult?	Open Response
Would you visit lakes and beaches in your area more often if swimmer's itch wasn't a concern?	Yes/No/The same amount
How did you hear about us?	Google search/Twitter/Flyer or handout/Information booth/Word of mouth/Community board posting/News from an internet site/News on the radio/News on Television/Other
If other, please list where you heard of us?	Open Response
Open-ended comments	Open Response

§ Question added in 2014
