# Supplementary material for: Swimmer’s itch in Canada: a look at the past and a survey of the present to plan for the future
Source: Environ Health. 2018 Oct 25;17:73. doi: 10.1186/s12940-018-0417-7 (PMC6203143; doi:10.1186/s12940-018-0417-7)
Supplement: Supplementary file 3 — Table S3. GLM Model Comparisons for Effects of Year and Month on Swimmer’s Itch Occurrences. The metrics within this table were used to compare deviance across models and to decide the best fit model. Abbreviations: Df/df = Degrees of Freedom, Resid. = residual, Dev. = Deviance, loglik = log likelihood, AIC = Akaike Information Criterion, BIC = Bayesian Information Criterion, dAIC = delta AIC. The best model was selected on the lowest AIC and BIC, having a residual deviance equal to the residual degrees of freedom, and a high log likelihood. The best model was therefore Model 1. (PDF 323 kb) [file 12940_2018_417_MOESM3_ESM.pdf]

	Resid. Df	Resid. Dev	Df	Deviance	Pr(>Chi)	logLik	AIC	BIC	dAIC	df	weight
**Model 1: cases ~ year * month**	0	-1.49E-14	NA	NA	NA	-58.0552	154.1105	172.0548	0	19	1
**Model 2: cases ~ month + year**	11	359.2	-11	-359.24	< 2.2e-16	-237.676	491.3527	498.9082	337.2	8	<0.001
**Model 3: cases ~ year**	15	3335	-4	-2975.77	< 2.2e-16	-1725.56	3459.125	3462.903	3305	4	<0.001
**Model 4: cases ~ month**	14	686.3	1	2648.69	< 2.2e-16	-401.217	812.434	817.1562	658.3	5	<0.001
