# Supplementary material for: Swimmer’s itch in Canada: a look at the past and a survey of the present to plan for the future
Source: Environ Health. 2018 Oct 25;17:73. doi: 10.1186/s12940-018-0417-7 (PMC6203143; doi:10.1186/s12940-018-0417-7)
Supplement: Supplementary file 4 — Table S4. Estimates of Evolutionary Distance Between Pairs of Groups of Trichobilharzia and other Avian Schistosomatid Species. The number of base differences per site from averaging over all sequence pairs between groups are shown. Standard error estimate(s) are shown above the diagonal. Within group divergence is shown on the diagonal and in bold. The range of within group divergence is in parentheses after the group name on the first column. The rate variation among sites was modeled with a gamma distribution (shape parameter = 1). The analysis involved 85 nucleotide sequences. Codon positions included were 1st + 2nd + 3rd + Noncoding. All positions containing gaps and missing data were eliminated. There were a total of 417 positions in the final dataset. Evolutionary analyses were conducted in MEGA7 [30]. (PDF 459 kb) [file 12940_2018_417_MOESM4_ESM.pdf]

**Species group (w/in group divergence range)**	*A. vari.*	*Avian sp.A*	*Avian sp.B*	*Avian sp.C*	*T. anseri*	*T. brantae*	*T. franki*	*T. mergi*	*T. phys.*	*T. quer.*	*T. regenti*	*T. sp. A*	*T. sp. B*	*T. sp. C*	*T. sp. D*	*T. sp. E*	*T. stag.*	*T. szidati*	W1285	W2081
A. variglandis	-	0.018	0.019	0.019	0.018	0.019	0.019	0.019	0.018	0.019	0.017	0.018	0.018	0.019	0.018	0.018	0.018	0.018	0.019	0.019
Avian sp. A (0.0-3.6%)	0.187	**0.011**	0.016	0.018	0.017	0.018	0.018	0.017	0.017	0.016	0.017	0.018	0.018	0.017	0.018	0.017	0.017	0.017	0.019	0.017
Avian sp. B	0.206	0.141	-	0.018	0.018	0.018	0.017	0.017	0.017	0.017	0.017	0.017	0.017	0.016	0.017	0.018	0.017	0.016	0.018	0.018
Avian sp. C	0.204	0.181	0.185	-	0.017	0.019	0.018	0.017	0.017	0.018	0.017	0.018	0.018	0.018	0.017	0.017	0.016	0.017	0.018	0.019
T. anseri (0.0-0.7%)	0.181	0.166	0.184	0.157	**0.002**	0.017	0.014	0.015	0.014	0.015	0.014	0.014	0.015	0.014	0.014	0.014	0.015	0.014	0.017	0.018
T. brantae (0.0%)	0.187	0.180	0.187	0.187	0.146	**0.000**	0.016	0.017	0.016	0.016	0.015	0.016	0.016	0.016	0.016	0.017	0.016	0.015	0.017	0.018
T. franki (0.0-1.7%)	0.197	0.169	0.171	0.175	0.116	0.141	**0.006**	0.015	0.012	0.012	0.014	0.013	0.012	0.012	0.015	0.015	0.015	0.014	0.017	0.017
T. mergi (0.0-0.7%)	0.182	0.175	0.176	0.150	0.112	0.153	0.115	**0.004**	0.015	0.014	0.012	0.016	0.014	0.015	0.014	0.015	0.015	0.013	0.017	0.017
T. physellae (0.0-1.0%)	0.185	0.175	0.163	0.160	0.116	0.141	0.074	0.119	**0.004**	0.012	0.014	0.013	0.012	0.013	0.015	0.016	0.015	0.014	0.018	0.017
T. querquedulae (0.2-2.6%)	0.206	0.158	0.171	0.171	0.125	0.133	0.072	0.110	0.081	**0.011**	0.014	0.013	0.012	0.012	0.014	0.015	0.015	0.013	0.016	0.017
T. regenti (0.0-3.6%)	0.185	0.175	0.175	0.154	0.119	0.120	0.110	0.089	0.106	0.109	**0.017**	0.014	0.013	0.014	0.014	0.013	0.014	0.013	0.015	0.017
T. sp. A (0.2-1.4%)	0.195	0.166	0.169	0.173	0.124	0.131	0.083	0.125	0.095	0.081	0.100	**0.010**	0.011	0.013	0.014	0.015	0.015	0.013	0.017	0.017
T. sp. B	0.187	0.173	0.175	0.165	0.129	0.141	0.071	0.104	0.078	0.068	0.086	0.058	-	0.012	0.014	0.015	0.015	0.013	0.016	0.016
T. sp. C (1.2%)	0.198	0.182	0.173	0.187	0.127	0.129	0.084	0.127	0.086	0.088	0.102	0.091	0.079	**0.012**	0.015	0.016	0.015	0.014	0.016	0.017
T. sp. D (0.0%)	0.177	0.186	0.165	0.149	0.120	0.127	0.114	0.098	0.119	0.111	0.103	0.107	0.103	0.120	**0.000**	0.014	0.014	0.013	0.017	0.017
T. sp. E (0.0-0.5%)	0.171	0.167	0.177	0.153	0.103	0.149	0.119	0.112	0.125	0.124	0.102	0.125	0.122	0.132	0.092	**0.003**	0.014	0.013	0.016	0.018
T. stagnicolae (0.0-5.5%)*	0.175	0.162	0.162	0.153	0.125	0.140	0.127	0.124	0.135	0.121	0.114	0.121	0.125	0.126	0.109	0.104	**0.013**	0.014	0.017	0.017
T. szidati (0.5-4.8%)	0.194	0.158	0.158	0.156	0.106	0.123	0.111	0.102	0.112	0.103	0.109	0.103	0.102	0.120	0.100	0.100	0.118	**0.030**	0.016	0.017
W1285	0.194	0.195	0.189	0.177	0.155	0.161	0.157	0.147	0.157	0.143	0.118	0.149	0.132	0.155	0.146	0.140	0.155	0.156	-	0.017
W2081	0.218	0.152	0.170	0.175	0.182	0.173	0.157	0.169	0.169	0.158	0.165	0.152	0.141	0.167	0.161	0.173	0.144	0.153	0.168	-

* excluding FJ174493, range is 0.0-1.4%
