# Supplementary figures and images for: Swimmer’s itch in Canada: a look at the past and a survey of the present to plan for the future
Source: Environ Health. 2018 Oct 25;17:73. doi: 10.1186/s12940-018-0417-7 (PMC6203143; doi:10.1186/s12940-018-0417-7)

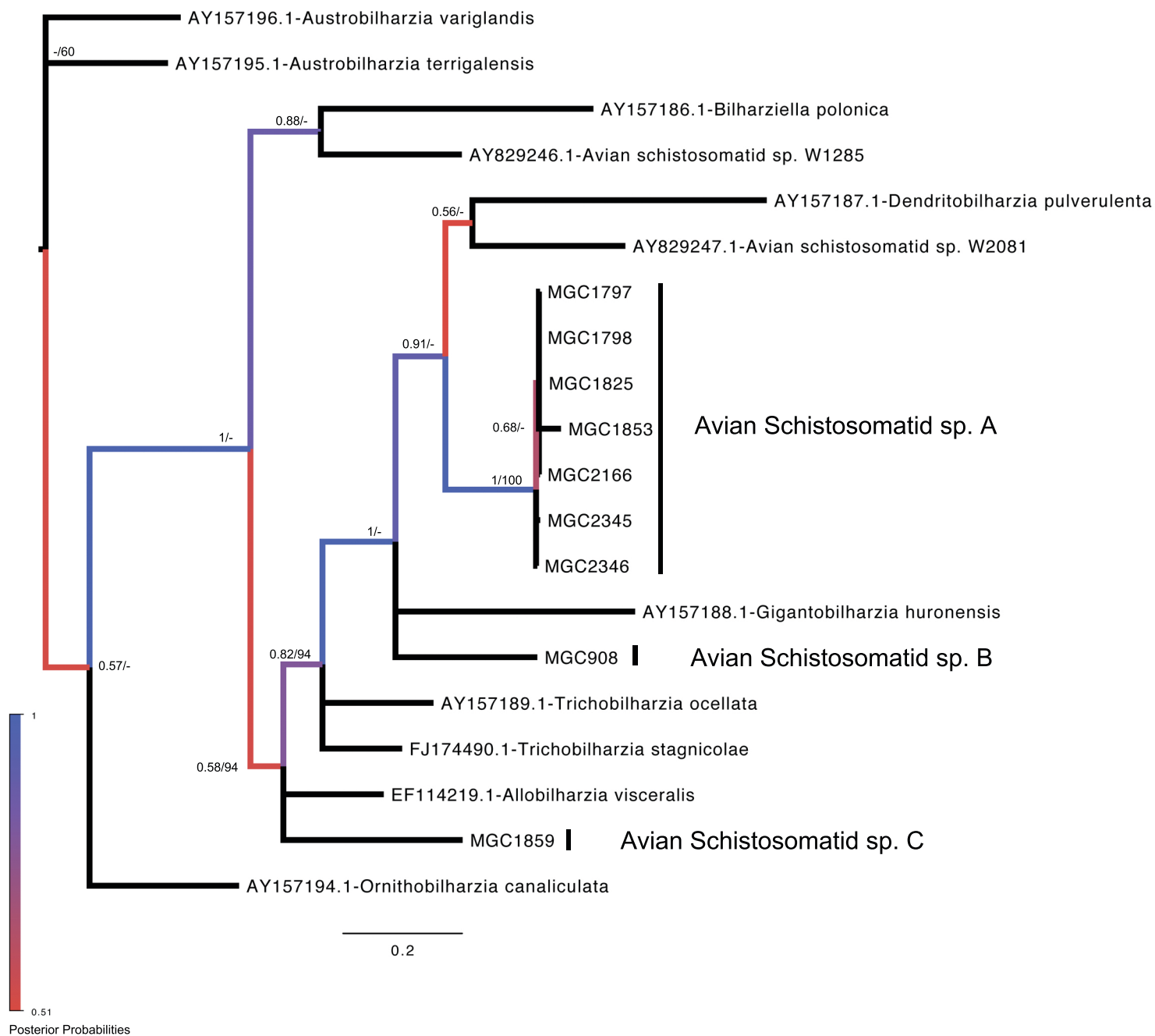

Supplement: Supplementary file 5 — Figure S1. Phylogenetic Tree of Avian Schistosomes. Tree topology is based on Bayesian Inference. Nodal support is indicated with posterior probabilities (shown by coloured branches and associated numeric probabilities) followed by bootstrap support from Maximum Likelihood. GenBank accession numbers precede taxon names. All sequences from this study are labeled with ‘MGC’. (PDF 15551 kb) [file 12940_2018_417_MOESM5_ESM.pdf]

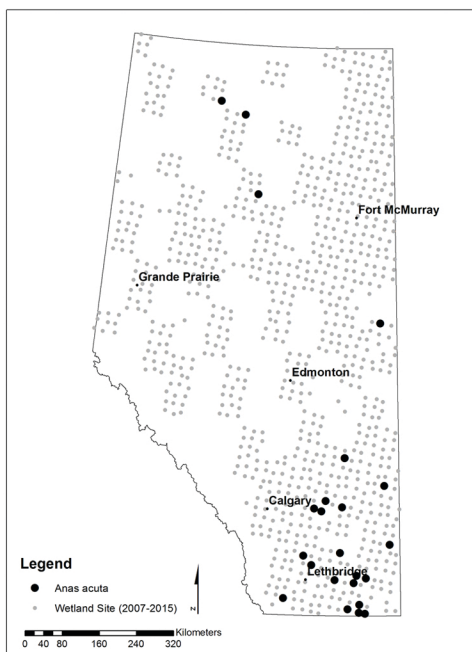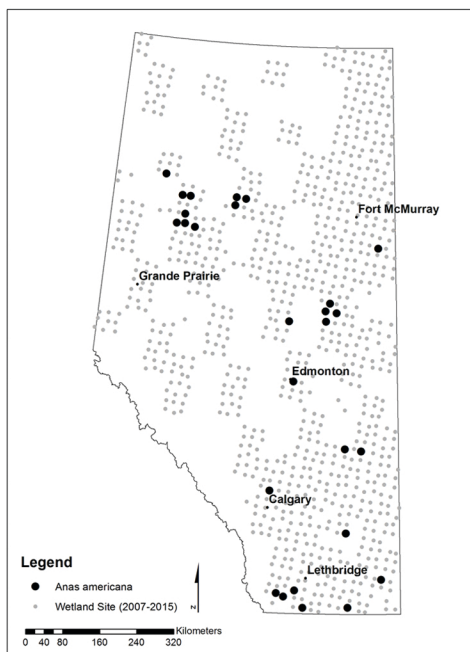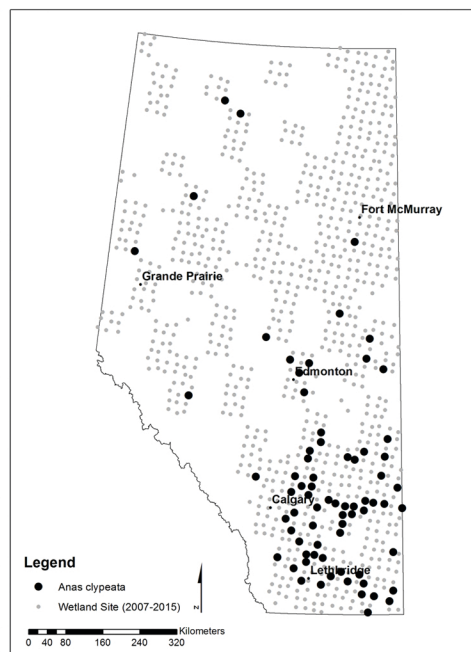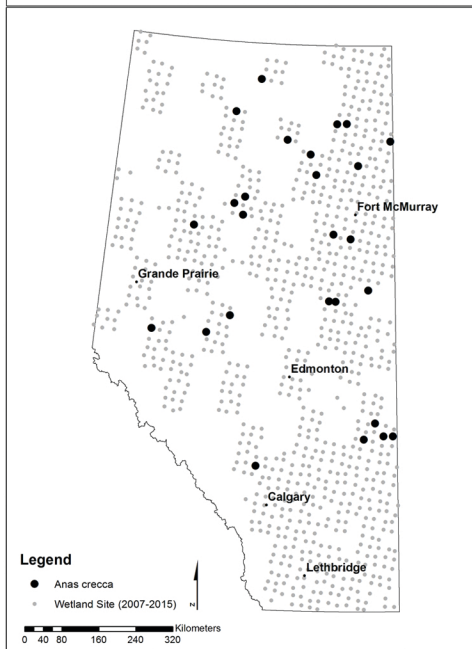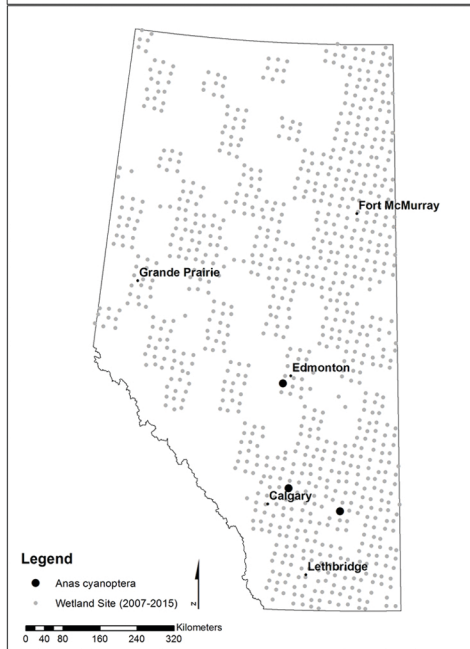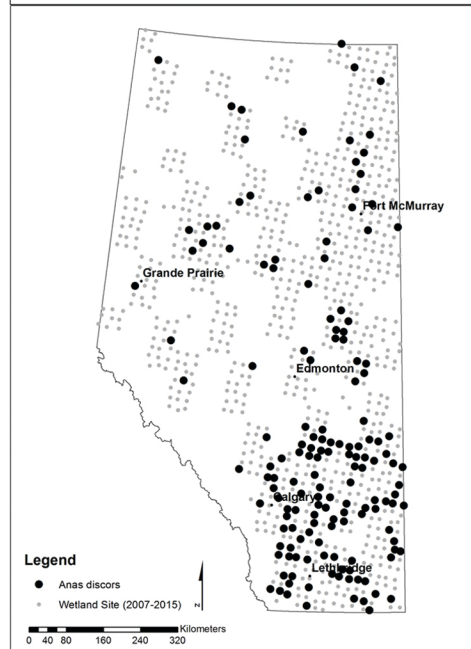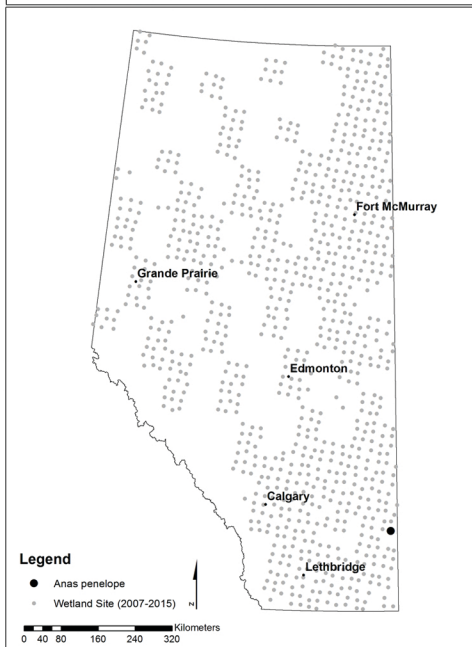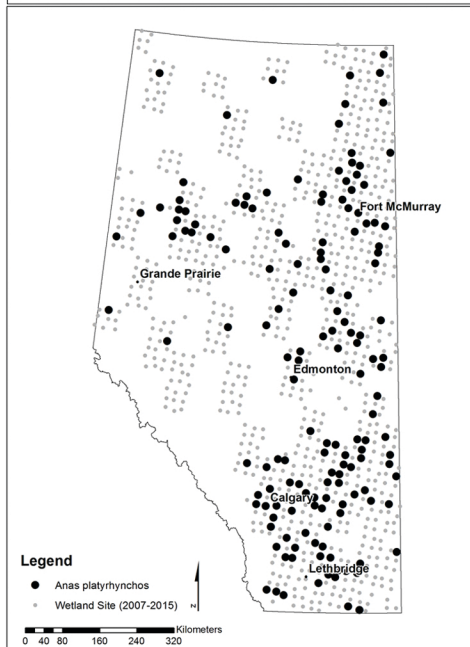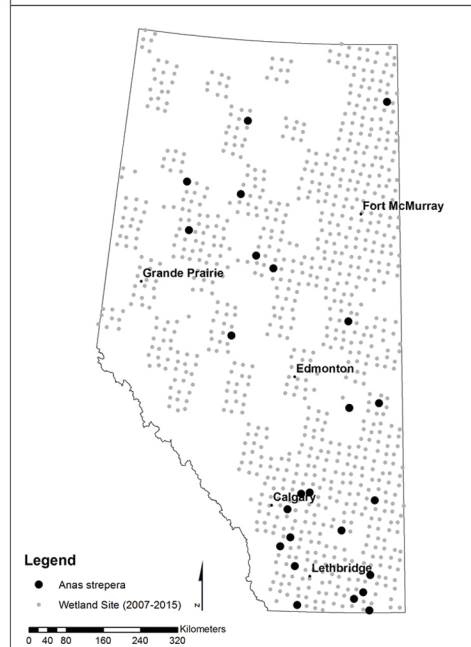

Supplement: Supplementary file 6 — Figure S2. Anas spp. Distributions Across Alberta Wetlands. Distributions of 9 Anas spp. (waterfowl/ducks) as collected by ABMI. (PDF 4716 kb) [file 12940_2018_417_MOESM6_ESM.pdf]

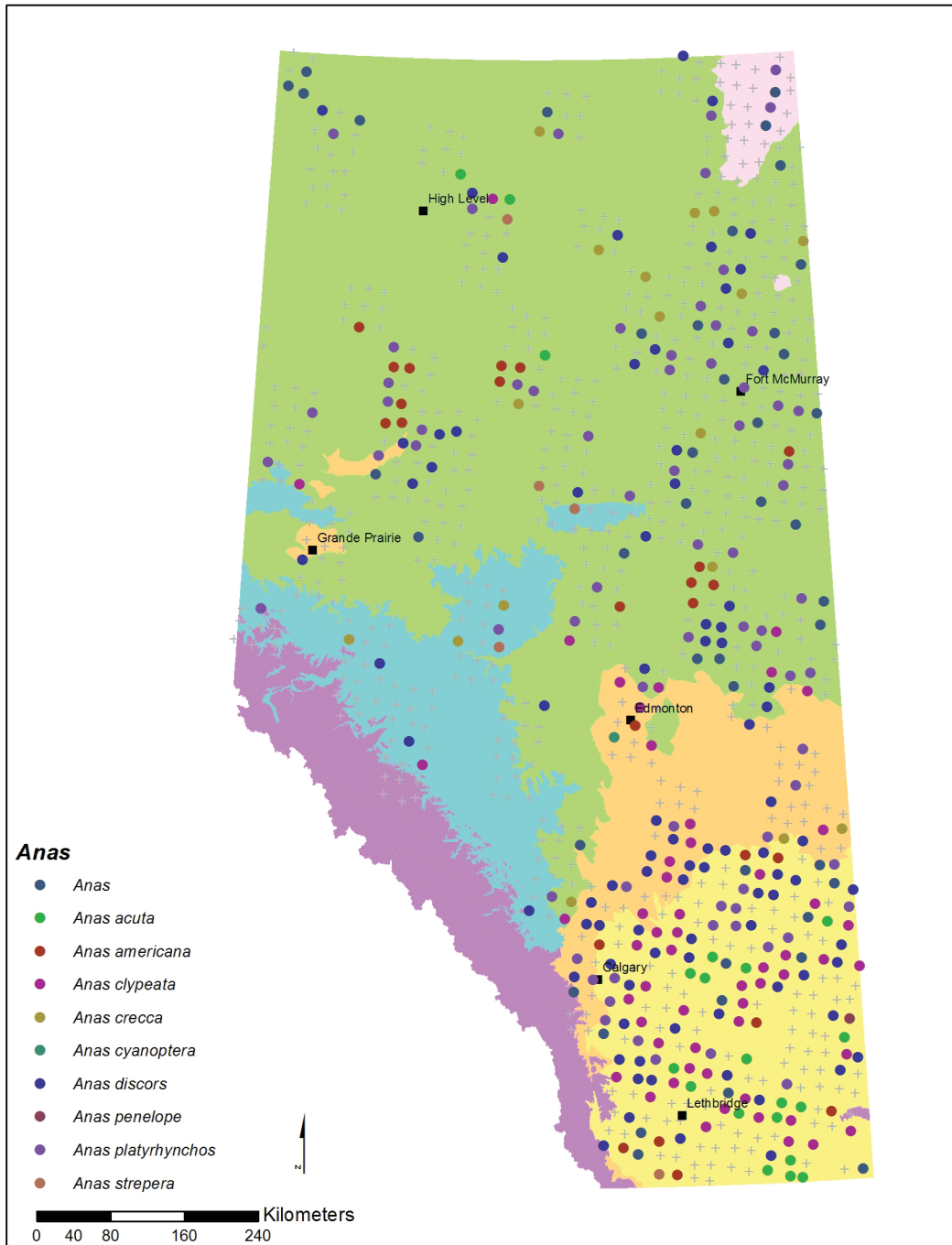

Supplement: Supplementary file 7 — Figure S3. Combined Distributions of Anas spp. Across Alberta Wetlands. (PDF 3152 kb) [file 12940_2018_417_MOESM7_ESM.pdf]

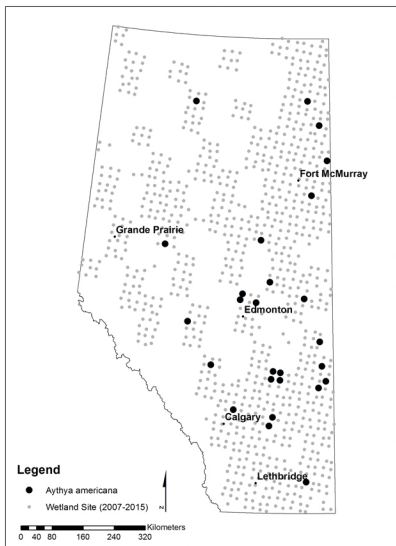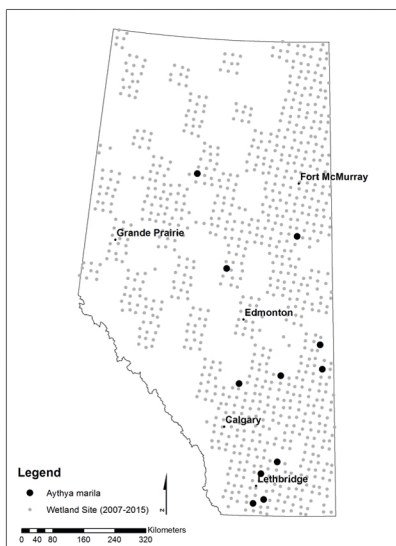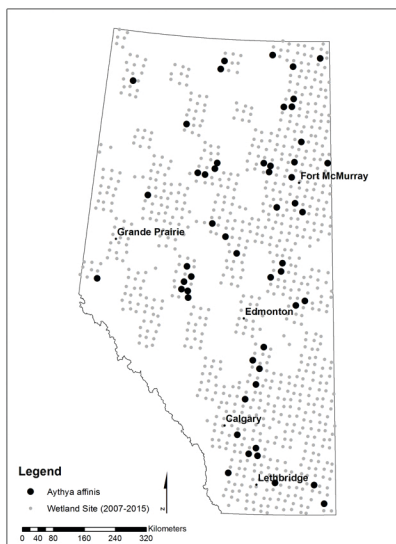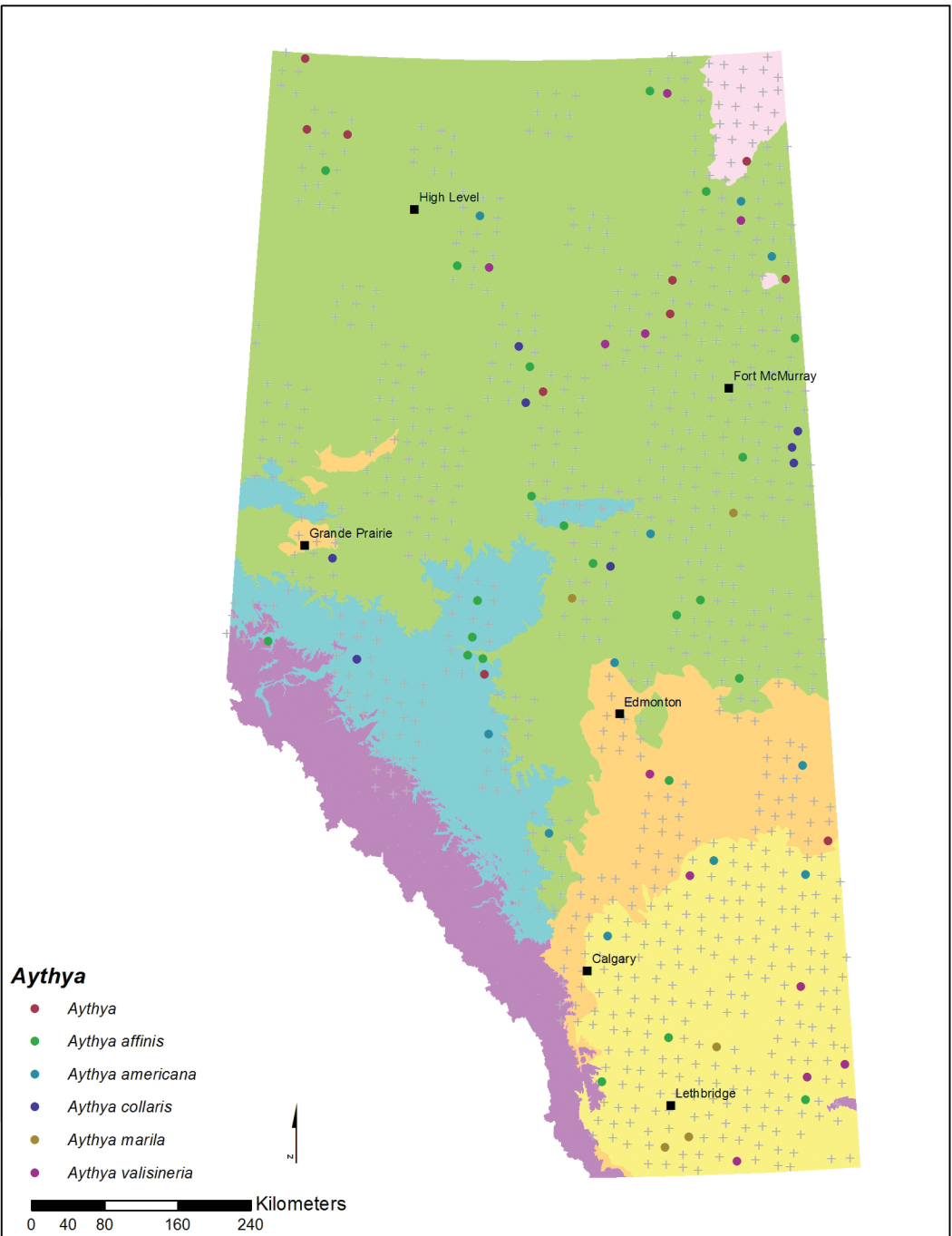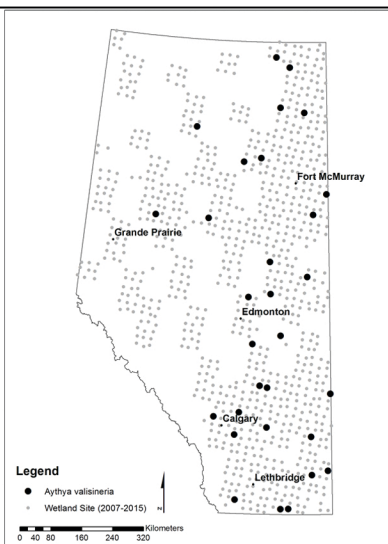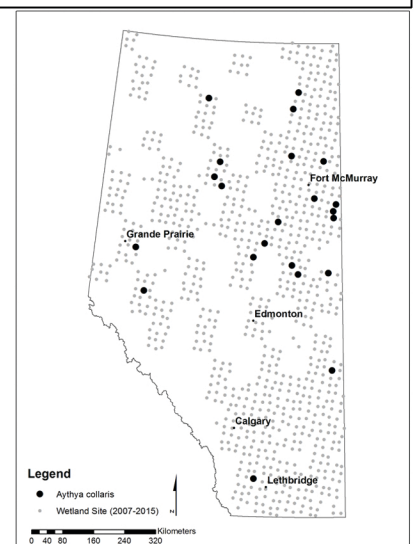

Supplement: Supplementary file 8 — Figure S4. Aythya spp. Distributions Across Alberta Wetlands. Distributions of 5 Aythya spp. (waterfowl/ducks) as collected by ABMI, both individual and in combination. (PDF 4377 kb) [file 12940_2018_417_MOESM8_ESM.pdf]

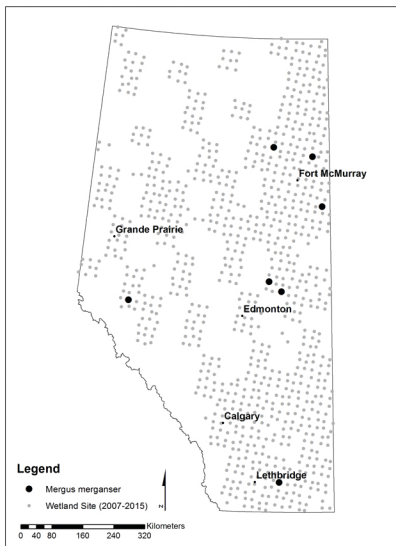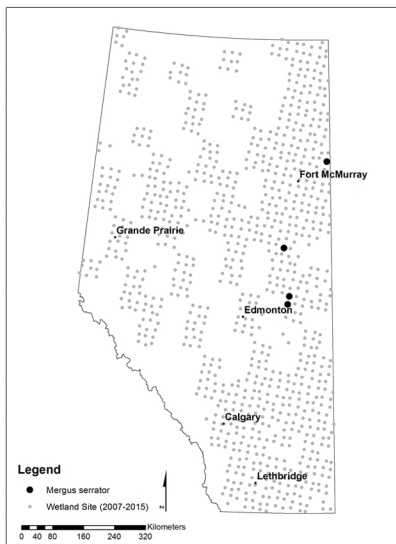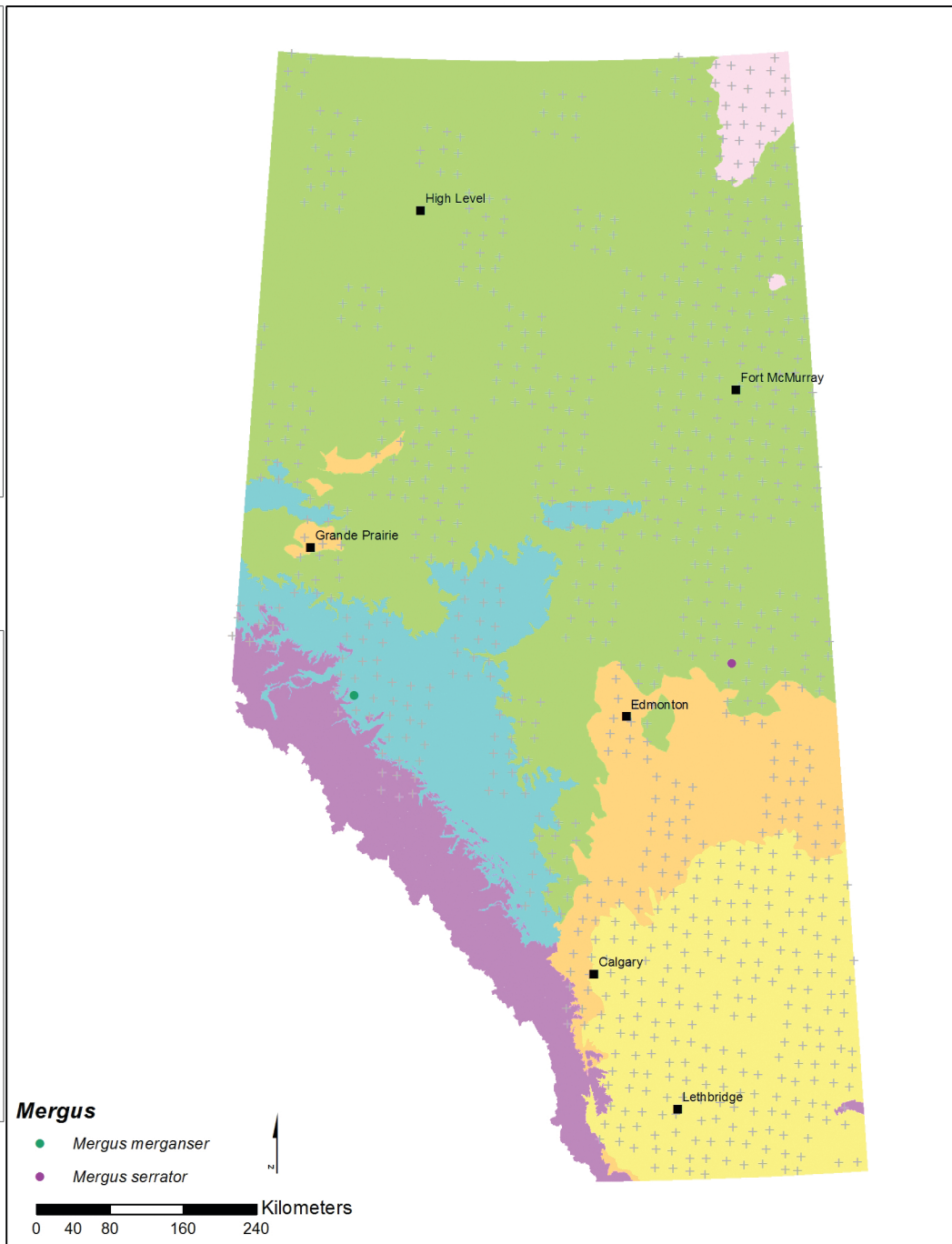

Supplement: Supplementary file 9 — Figure S5. Merganser spp. Distributions Across Alberta Wetlands. Distributions of 2 species of diving ducks as collected by ABMI. Both individual and combined distributions are reported. (PDF 3281 kb) [file 12940_2018_417_MOESM9_ESM.pdf]

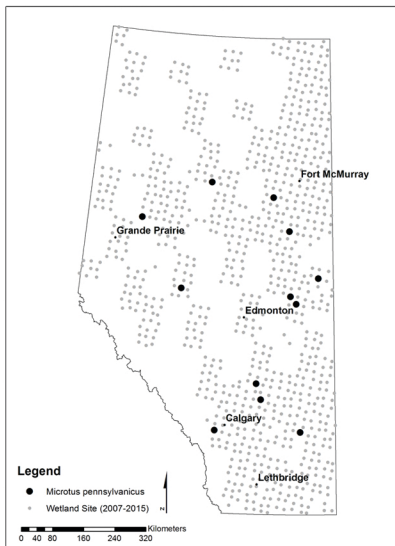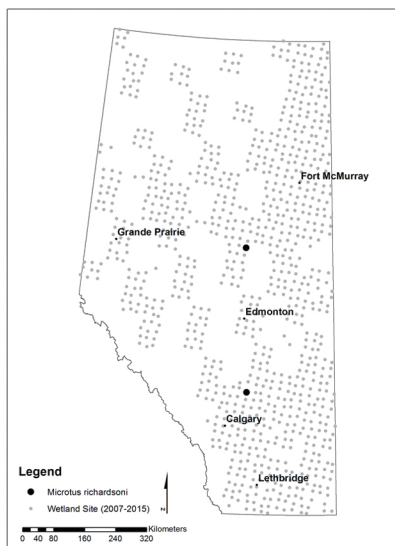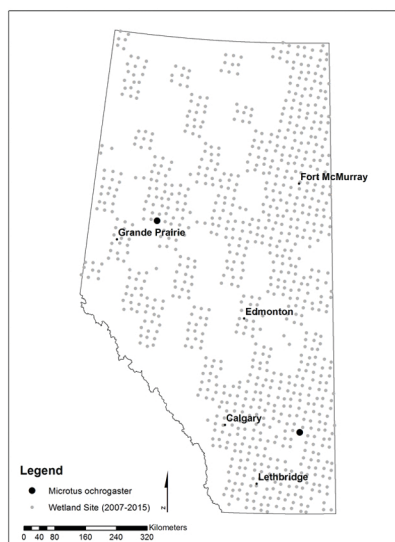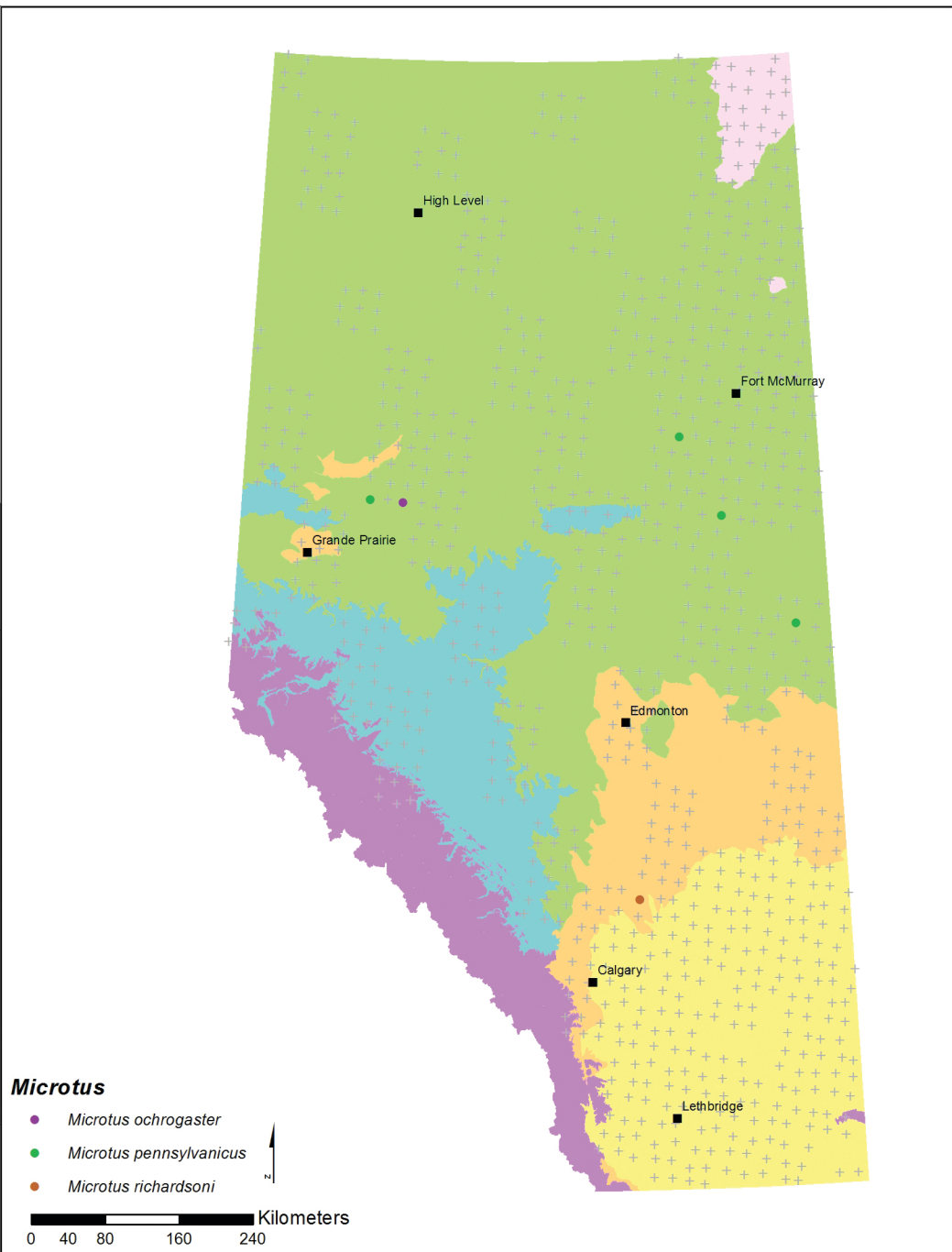

Supplement: Supplementary file 10 — Figure S6. Microtus spp. Distributions Across Alberta Wetlands. Distributions of 3 species of Muskrat as collected by ABMI. Both individual and combined distributions are reported. (PDF 3594 kb) [file 12940_2018_417_MOESM10_ESM.pdf]

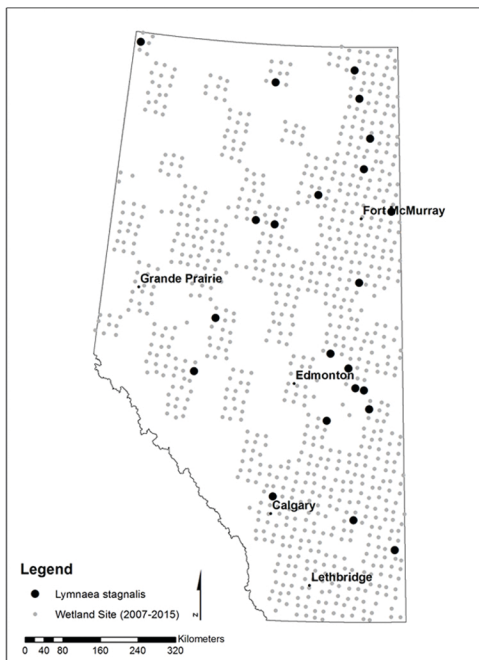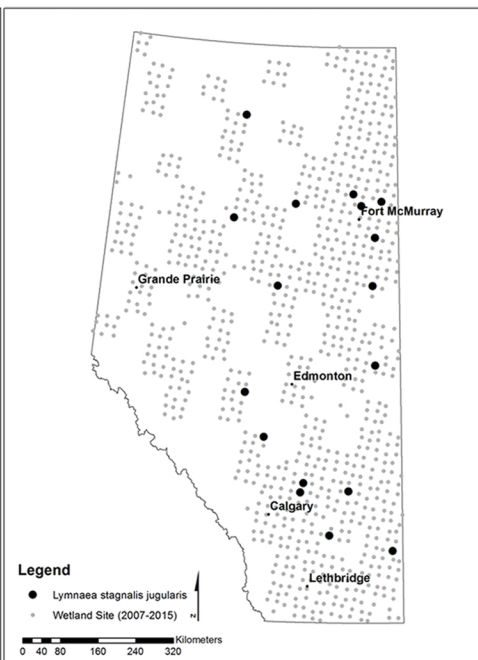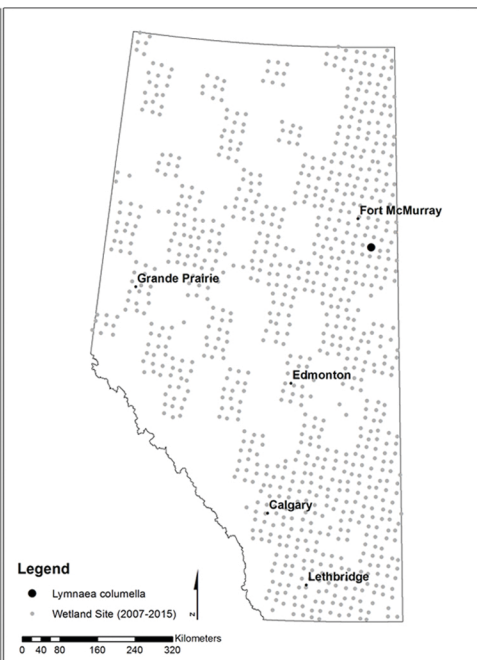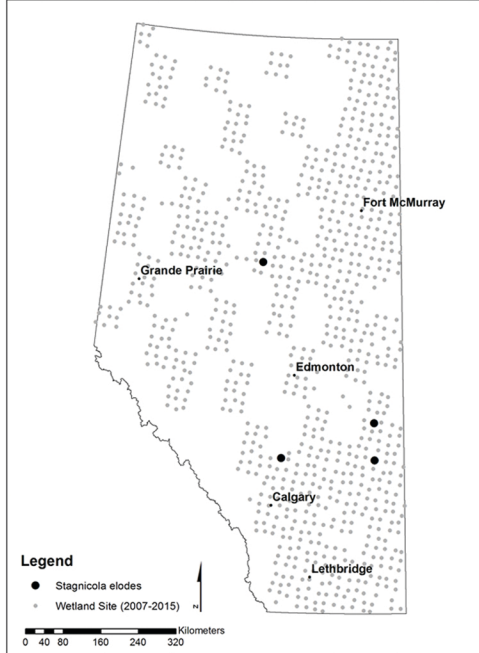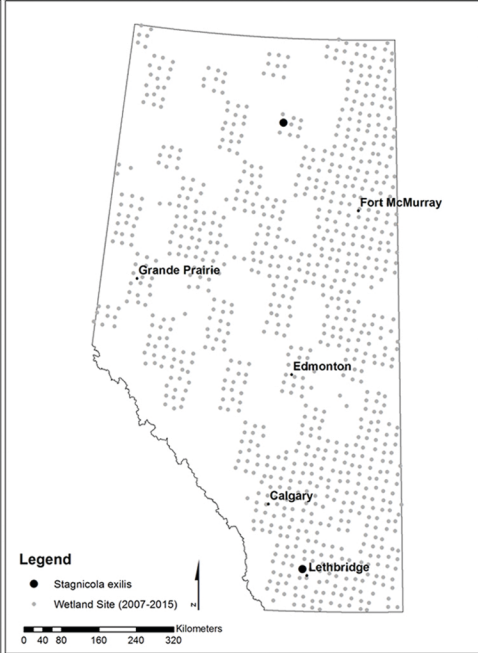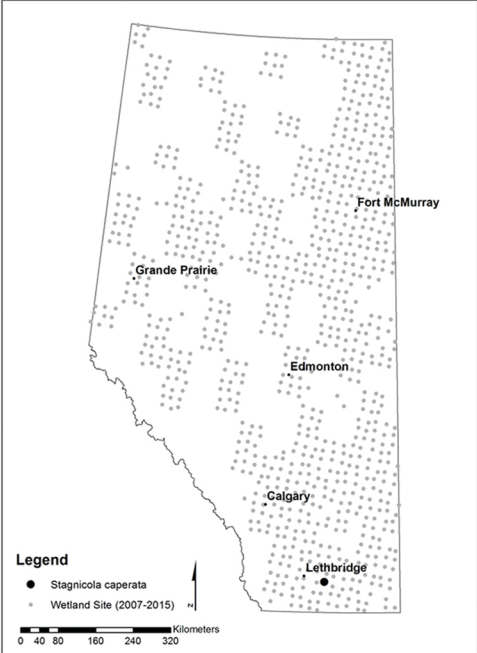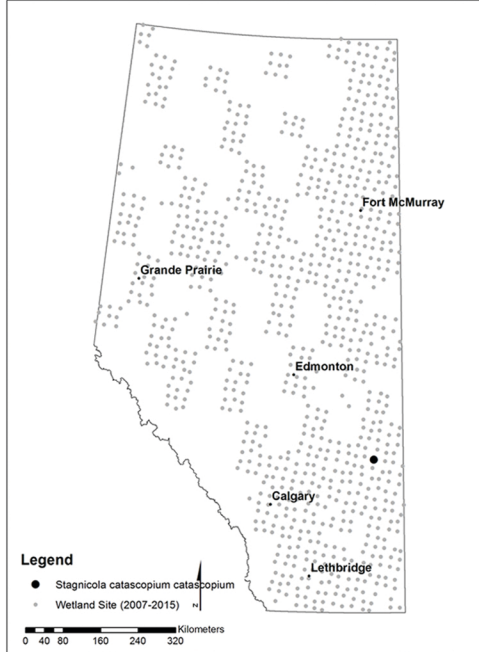

Supplement: Supplementary file 11 — Figure S7. Lymnaeid Snail Distributions Across Alberta Wetlands. (PDF 3580 kb) [file 12940_2018_417_MOESM11_ESM.pdf]

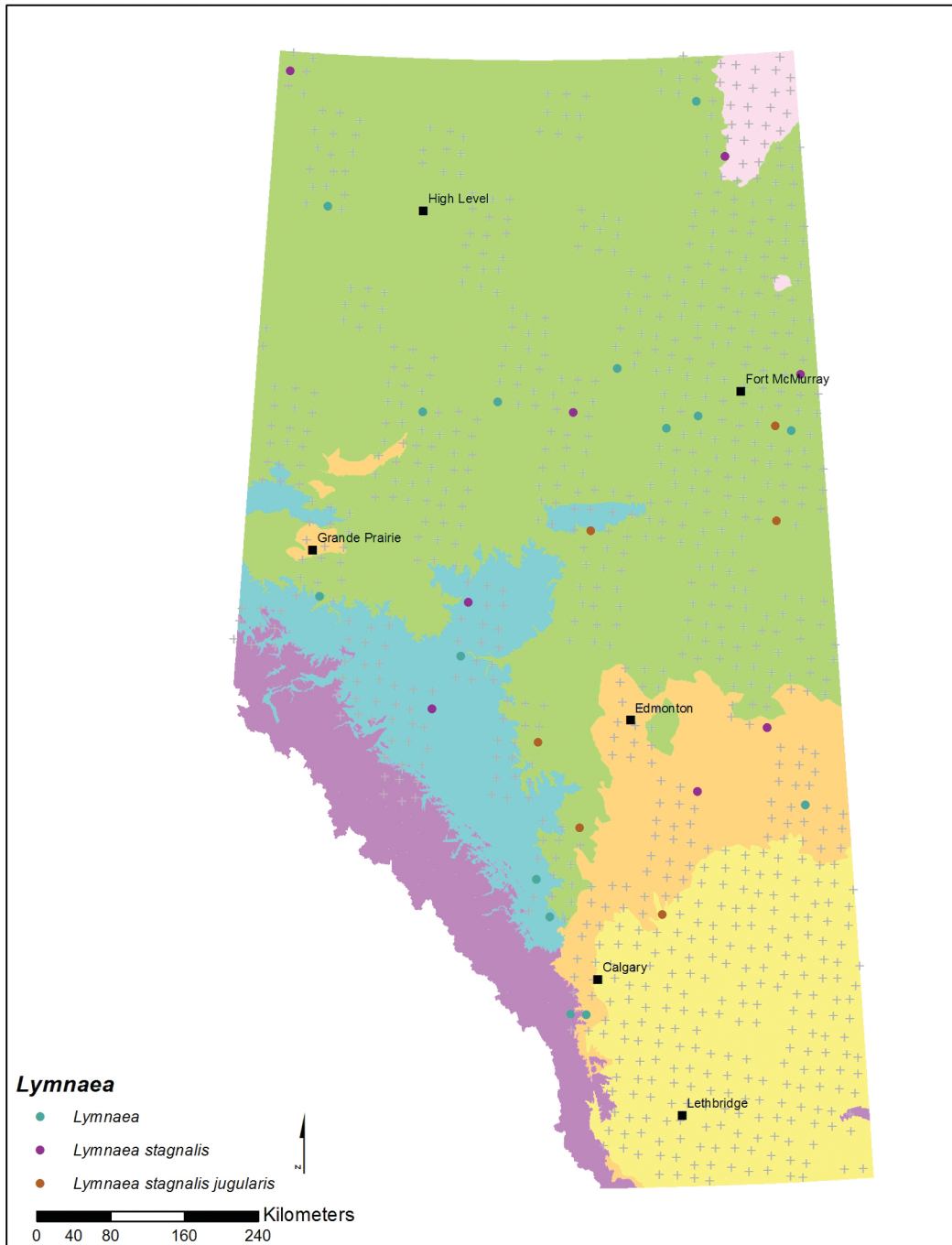

Supplement: Supplementary file 12 — Figure S8. Combined Lymnaeid Snail Distributions Across Alberta Wetlands. (PDF 2768 kb) [file 12940_2018_417_MOESM12_ESM.pdf]

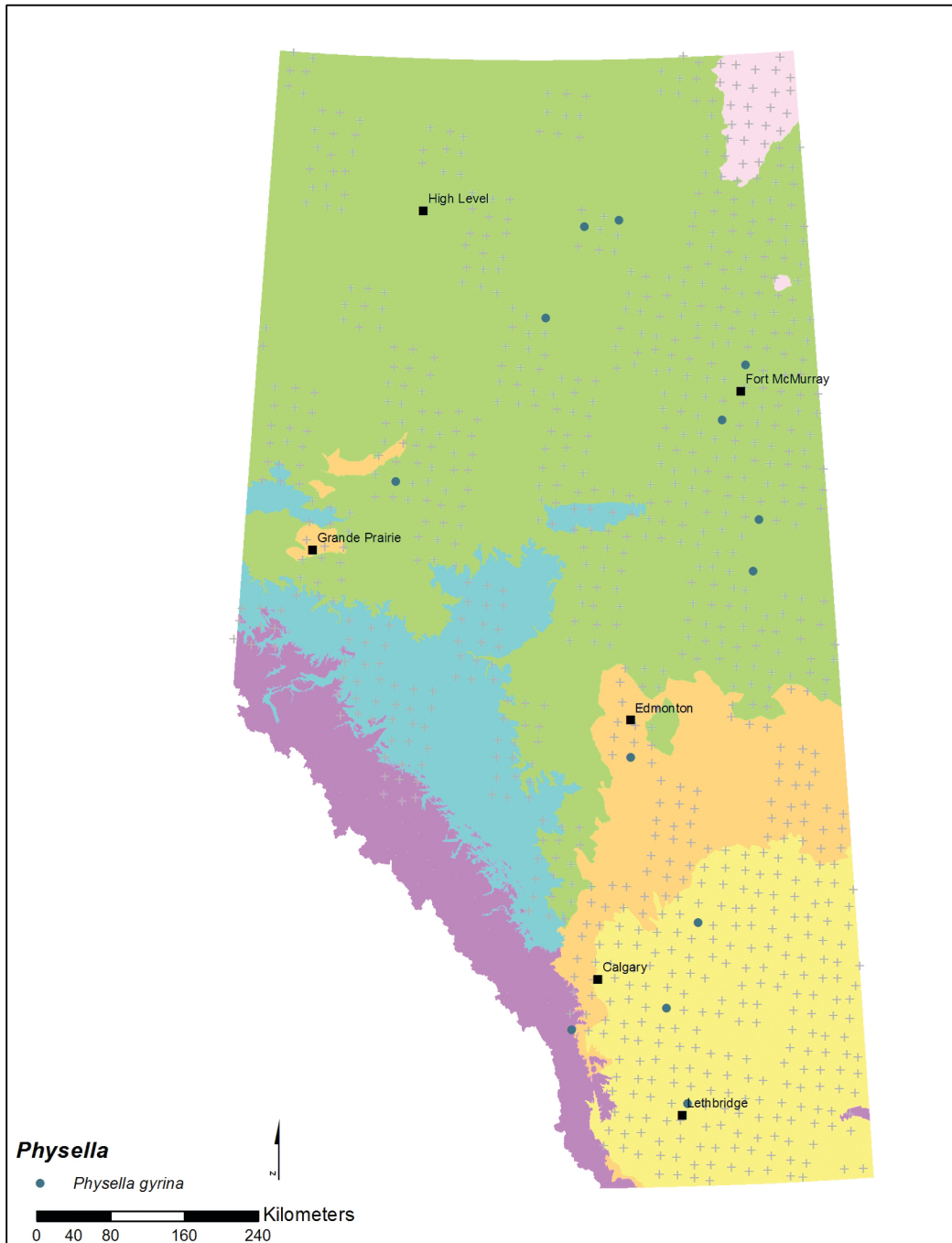

Supplement: Supplementary file 13 — Figure S9. Physid Snail Distributions Across Alberta Wetlands. (PDF 2720 kb) [file 12940_2018_417_MOESM13_ESM.pdf]

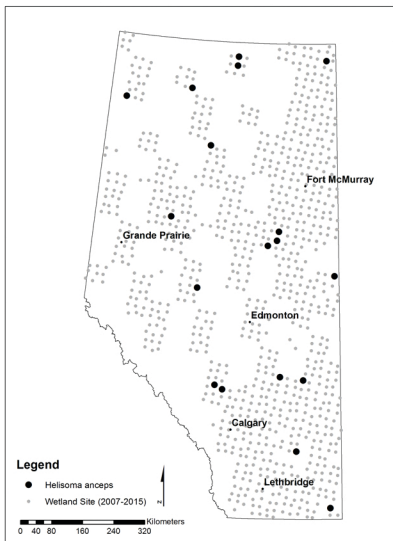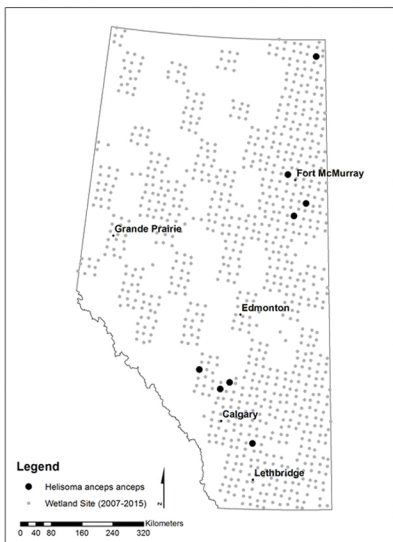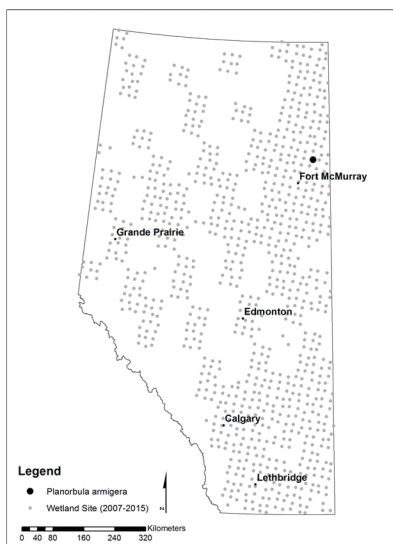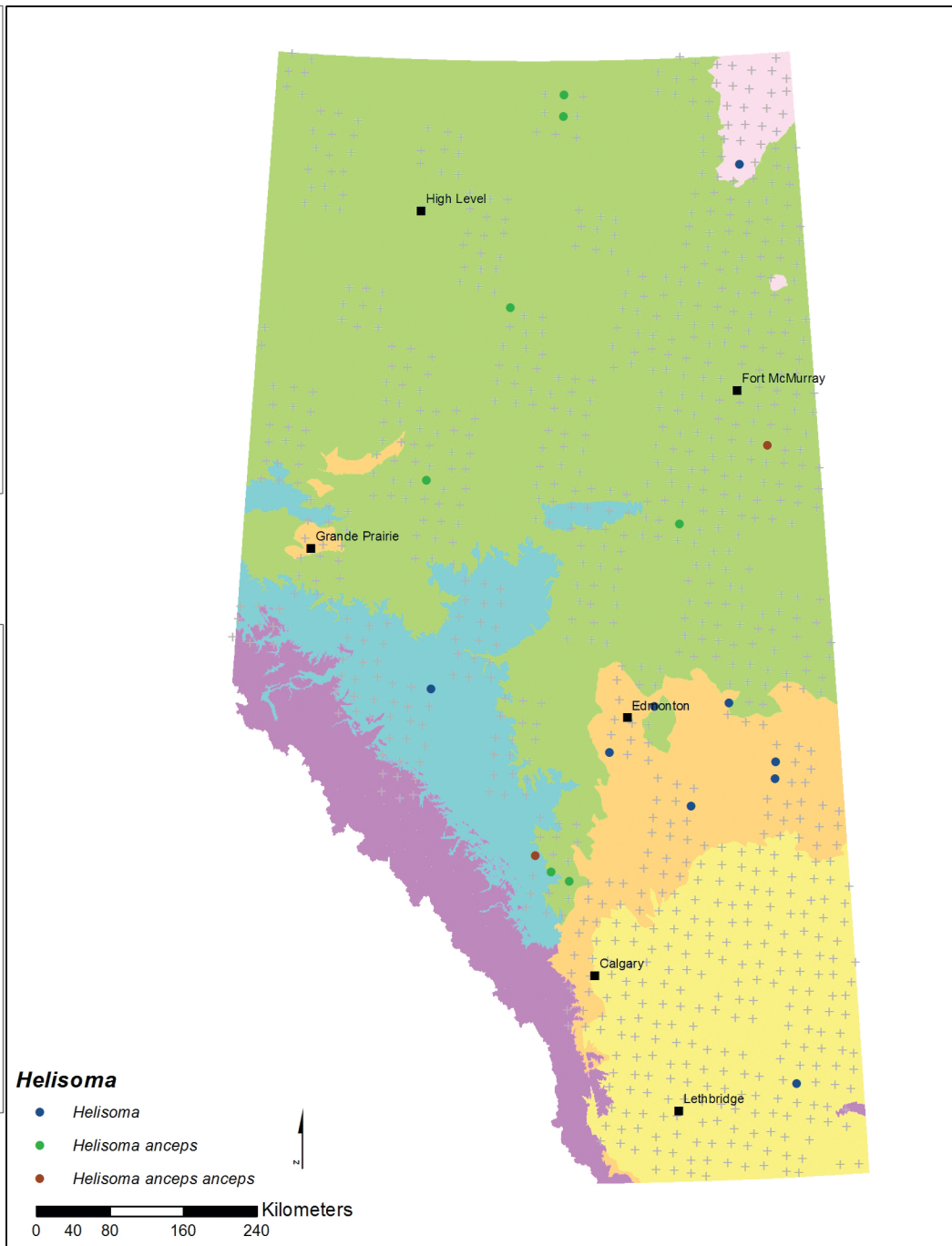

Supplement: Supplementary file 14 — Figure S10. Planorbidae Snail Distributions Across Alberta Wetlands. (PDF 3638 kb) [file 12940_2018_417_MOESM14_ESM.pdf]
