# Supplementary material for: Swimmer’s itch in Canada: a look at the past and a survey of the present to plan for the future
Source: Environ Health. 2018 Oct 25;17:73. doi: 10.1186/s12940-018-0417-7 (PMC6203143; doi:10.1186/s12940-018-0417-7)
Supplement: Supplementary file 15 — Table S5. Lake Locations of Swimmer’s Itch Reports. (PDF 368 kb) [file 12940_2018_417_MOESM15_ESM.pdf]

Province	Lake	Cases	Years Reported
Alberta	Athabasca River at Whispering Hills	2	2015
	Baptiste Lake	1	2016
	Barrier Lake	1	2016
	Battle Lake	1	2015
	Bear Lake	8	2014
	Bear Trap Lake	13	2015
	Bearhills Lake	1	2013
	Bellis Beach Lake	1	2013
	Buck Lake	14	2015, 2016, 2017
	Buffalo Lake	423	2013, 2014, 2015, 2016, 2017
	Burnstick Lake	1	2013
	Calling Lake	2	2013, 2017
	Cameron Lake	29	2015, 2017
	Capt. Ayr Lake	1	2013
	Chestermere Lake	5	2014, 2015
	Chickenhill Lake	7	2016, 2017
	Chump Lake	10	2013, 2015, 2016
	Cold Lake	55	2013, 2014, 2015, 2016
	Cornwall Lake	1	2013
	Cow Lake	2	2013
	Crimson Lake	2	2013
	Dilberry Lake	18	2015, 2017
	Edith Lake	15	2015, 2017
	Elkwater Lake	2	2016
	Fickle Lake	9	2015, 2016
	Floatingstone Lake	72	2013, 2015, 2016, 2017
	Fork Lake	5	2013, 2015
	Fox Creek Trout Pond	12	2014
	Garner Lake	39	2015, 2016
	Gerharts Lake	4	2015
	Ghost Lake	1	2014
	Granum Pond	1	2013
	Gregoire Lake	3	2013, 2015
	Gull Lake	15	2014, 2016, 2017
	Half Moon Lake	39	2013, 2014, 2015, 2016, 2017
	Hanmore Lake	69	2013, 2014, 2015, 2016
	Hasse Lake	1	2017
	Hermitage Pond	1	2013
	Hope Lake	33	2013, 2014, 2015, 2017
	Hubbles Lake	58	2013, 2015, 2016, 2017
	Island Lake	15	2015, 2016, 2017
	Jackfish Lake	49	2013, 2015, 2016, 2017
	Jarvis Lake	2	2014
	Kirk Lake	1	2017
	Lac Bellevue	24	2014, 2015
	Lac La Biche	14	2013, 2014, 2015, 2016

Lac la Nonne	1	2014
Lac Sante	45	2015, 2016, 2017
Lac St Cyr	4	2015
Lac Ste. Anne	14	2013, 2014, 2015, 2016
Lake Annette	1	2013
Lake Bonavista	16	2014, 2015
Lake Isle	4	2013, 2015
Lake Newell	1	2017
Laurier Lake	9	2013, 2015
Lessard Lake	4	2013
Lesser Slave Lake	11	2013, 2014, 2015, 2017
Little Bow Lake	4	2014, 2015
Long Island Lake	1	2013
Long Lake	62	2013, 2014, 2015, 2016, 2017
Lower Therien Lake	2	2014
Marie Lake	4	2013, 2014
McKenzie Lake	8	2015
Millers Lake	3	2017
Mink Lake	3	2015
Miquelon Lake	11	2015, 2016
Mons Lake	3	2015, 2017
Moose Lake	19	2013, 2014, 2015
Nakamun Lake	16	2013, 2015, 2016
North Buck Lake	61	2013, 2014, 2015, 2016, 2017
Open Creek Dam	2	2016
Park Lake	7	2015, 2017
Pigeon Lake	90	2013, 2014, 2015, 2016, 2017
Pine Lake	7	2017
Rattlesnake Lake	3	2013, 2017
Red Deer River	2	2015
Reesor Lake	4	2015
Rock Lake	1	2017
Ross Lake	16	2013, 2014, 2017
Rundle Park	1	2015
Saskatoon Lake	1	2013
Shorncliffe Lake	15	2013, 2015
Skeleton Lake	8	2016, 2017
Spring Lake	28	2013, 2014, 2015, 2016, 2017
Spruce Coulee Reservoir	1	2017
St Mary Reservoir	1	2015
Sundance Lake	1	2013
Sylvan Lake	25	2013, 2015, 2016, 2017
Three Mile Bend at Red Deer River	5	2016
Thunder Lake	8	2013, 2015
Touchwood Lake	4	2014
Travers Reservoir	2	2013, 2015
Trestle Creek Golf Resort	31	2015, 2016

	Twin Lake	44	2016, 2017
	Wabamun Lake	201	2013, 2014, 2015, 2016, 2017
	Wasa Lake	1	2015
	Wedge Pond	2	2017
	Whitefish Lake	4	2013, 2016
	Whitney Lake	26	2013, 2014, 2015, 2016, 2017
	Wizard Lake	3	2015, 2016
	Wolf Lake	10	2013, 2015
British Columbia	Adams Lake	24	2013, 2014, 2016, 2017
	Alta Lake	27	2013, 2014, 2015, 2017
	Arrow Lake Park	1	2013
	Arrow Lakes	1	2013
	Babine Lake	2	2013, 2016
	Bear Lake	3	2013, 2017
	Buttle Lake	12	2013, 2014, 2016, 2017
	Centennial Beach at Boundary Bay	1	2017
	Charlie Lake	3	2013
	Chehalis Lake	4	2015
	Chilliwack Lake	3	2013, 2016
	Columbia River	1	2013
	Comox Lake	10	2013, 2014, 2016, 2017
	Cowichan Lake	5	2015
	Crescent Beach	45	2013, 2014, 2015, 2016, 2017
	Cultus Lake	132	2013, 2014, 2015, 2016, 2017
	Cusheon Lake	5	2015
	Dunn Lake	7	2014
	Echo Lake	2	2015
	Enid Lake	1	2013
	Fishblue Lake	6	2016, 2017
	Francois Lake	2	2014
	Harrison Lake	29	2015, 2016, 2017
	Horne Lake	85	2013, 2014, 2015, 2016, 2017
	Inzana Lake	6	2017
	Kalamalka Lake	18	2013, 2014, 2015, 2016
	Kennedy Lake	3	2015
	Kin Beach	1	2013
	Kokanee Lake	1	2013
	Kootenay Lake	20	2013, 2014, 2017
	Langford Lake	4	2017
	Lac La Hache	12	2013, 2016, 2017
	Lake Pinantan	6	2015, 2017
	Lake Windermere	29	2013, 2014, 2016, 2017
	Little Shuswap Lake	17	2013, 2014, 2015
	Long Lake	1	2015
	Loon Lake	1	2014
	Lost Lake	1	2013
	Lost Lake (Whistler)	3	2017

	Mabel Lake	33	2013, 2014, 2015, 2016, 2017
	Madden Lake	1	2013
	Maiden Lake	4	2014
	Mara Lake	10	2013, 2016
	Monte Lake	2	2013, 2016
	Nadsilnich Lake	1	2017
	Nanaimo Lakes #2	1	2013
	Nicola Lake	4	2015
	Nukko Lake	2	2016
	Okanagan Lake	57	2013, 2014, 2015, 2016, 2017
	Osoyoos Lake	3	2013, 2015
	Pacific Ocean near White Rock	7	2013, 2014, 2016
	Paul Lake	20	2014, 2015, 2017
	Peckhams Lake	2	2015
	Premier Lake	1	2013
	Rolley Lake	7	2017
	Sasamat Lake	3	2013, 2016, 2017
	Shuswap Lake	281	2013, 2014, 2015, 2016, 2017
	Shuswap River	1	2014
	Spider Lake	3	2015
	Stuart Lake	1	2013
	Surveyors Lake	8	2013
	Thetis Lake	1	2017
	Tie Lake	2	2017
	Trout Lake	6	2014, 2015
	Wasa Lake	65	2013, 2014, 2015, 2016, 2017
	Weston Lake	4	2015
	Wood Lake	6	2015, 2017
	Woss Lake	2	2014
Manitoba	Clear Lake	23	2013, 2015, 2016
	Dorothy Lake	3	2017
	Kenton Reservoir	4	2015
	Lake Winnipeg	1	2017
	West Hawk Lake	1	2017
	Wild Oaks Campground Beach	21	2017
New Brunswick	Fisher Lakes	4	2015
	St John River	9	2014, 2015
Newfoundland	Northwest Pond	6	2013
Northwest Territories	Great Slave Lake	1	2013
Nova Scotia	Grand Lake	1	2013
	Lake Ainslie	4	2015
	Lake Banook	1	2015
	Mattatall Lake	1	2015
Ontario	Adams Lake	1	2016
	Balsam Lake	1	2017
	Baptiste Lake	1	2015
	Bass Lake	22	2013, 2015, 2016

Bass Lake near Lombardy	1	2013
Baxter Lake	3	2015
Bear Lake	3	2016
Berford Lake	1	2013
Big Rideau Lake	3	2016
Black River	2	2016
Bobs Lake	1	2013
Bon Echo Provincial Park at Mazinaw Lake	2	2016
Boshkung Lake	5	2016, 2017
Cameron Lake	3	2016
Centennial Lake	1	2013
Chesley Lake	2	2015
Commando Lake	3	2015
Cranberry Lake	1	2015
Crotch Lake	4	2015
Elliot Lake	1	2014
Farlain Lake	4	2017
Fitzroy Provincial Park	1	2013
Golden Lake	30	2013, 2015, 2016, 2017
Guelph Lake	1	2015
Gull Lake	5	2015
Gullivers Lake	9	2016
Harmony Beach at Lake Superior	2	2016
Havilland Bay at Lake Superior	3	2016
Horseshoe Lake	3	2014, 2016
Jack Lake	11	2013, 2014, 2017
Kashagawigamog Lake	1	2013
Kawawaymog Lake	1	2015
Kennisis Lake	1	2013
Koshlong Lake	1	2015
Lake Clear	14	2015, 2016
Lake Couchiching	2	2015
Lake Erie	15	2013, 2015, 2017
Lake Huron at Southampton	2	2015
Lake Huron Georgian Bay	21	2014, 2015, 2016, 2017
Lake Huron Sauble Beach	3	2013, 2017
Lake Kamaniskeg	11	2013
Lake Louisa	2	2016
Lake Manitou	1	2013
Lake Muskoka	5	2015
Lake Nipissing	30	2013, 2014, 2015, 2016, 2017
Lake Nosbonsing	6	2017
Lake Ontario	5	2013, 2016, 2017
Lake Rosseau	1	2014
Lake Temagami	2	2016
Lake Wilcox	1	2017
Lake Wolsey	2	2015

	Limerick Lake	2	2015
	Little Cranberry Lake	3	2015
	Marl Lake	3	2015
	Mazinaw Lake	16	2013, 2015, 2016
	Mink Lake	3	2016
	Missinaibi Lake	3	2017
	Mississippi River	11	2016
	Moon River at Georgian Bay	1	2016
	Mooneys Bay	1	2013
	Musselman Lake	4	2017
	Napanee River (Camden East)	2	2017
	Orr Lake	9	2015, 2016
	Ottawa River at Harvey Creek	4	2015
	Ottawa River at Haydon Park	1	2015
	Ottawa River Pembroke	2	2015
	Peninsula Lake	2	2016
	Pigeon Lake	1	2013
	Red Cedar Lake	3	2015
	Regina Bay at Glouster Pool at Georgian Bay	3	2016
	Remi Lake	4	2014, 2015
	Rice Lake	2	2013
	Riley Lake	3	2015
	Round Lake	2	2017
	Sandbar Lake	2	2017
	Shabomeka Lake	8	2015, 2016
	Simcoe Lake	15	2013, 2015, 2016
	Six Mile Lake	3	2016
	Spencer Creek	1	2013
	St Marys River	1	2013
	Stoney Lake	1	2017
	Sturgeon Bay Provincial Park at Georgian Bay	2	2016
	Tait Lake	1	2016
	Thunder Bay at Lake Superior	17	2016
	Trout Lake	30	2013, 2014, 2015, 2016
	Upper Canada Campground Pond	10	2014
	Valens Lake	5	2016
	Wasaga Beach Georgian Bay	2	2015
	White Lake	4	2014, 2015
	Wild Goose Beach Lake Superior	3	2015
	Wollaston Lake	1	2016
Quebec	Grand Lac MacDonald/Lake MacDonald	5	2013, 2016
	Lac Cameron	1	2013
	Lac des Seize Iles	1	2013
	Lac Massawippi	1	2016
	Lac Meech	2	2016
	Lac Opasatica	1	2013
	Lac Phillipe	5	2013, 2016

	Riviere des Outaouais	3	2014
Saskatchewan	Blackstrap Lake	3	2015
	Buffalo Pound Lake	6	2013, 2015
	Candle Lake	3	2015
	Chitek Lake	18	2017
	Delaronde Lake	1	2015
	Greenwater Lake	2	2013
	Greig Lake	54	2013, 2014, 2015, 2016, 2017
	Jackfish Lake	22	2016
	Jeannette Lake	1	2013
	Jumbo Lake	1	2013
	Kimball Lake	36	2014, 2015, 2016, 2017
	Lac des Iles	5	2013, 2017
	Lac La Ronge	1	2013
	Last Mountain Lake	2	2017
	Lower Fishing Lake	3	2013
	Madge Lake	8	2013, 2015
	Makwa Lake	1	2013
	Marean Lake	2	2013
	Martins Lake	51	2017
	Matheson Lake	16	2015, 2016
	Meeting Lake	1	2013
	Memorial Lake	7	2013, 2016, 2017
	Murray Lake	1	2017
	Pierce Lake	28	2013, 2015, 2016
	Shell Lake	3	2013, 2014
	Suffern Lake	16	2013, 2014, 2017
	Turtle Lake	10	2014, 2017
	Wakaw Lake	6	2013, 2015
	Waskesiu Lake	3	2016, 2017
United States	Detroit Lake	1	2015
	Frenchman Lake	3	2016
	Higgins Lake	1	2013
	Lake McDonald	2	2013
	Osoyoos Lake	2	2015
	San Diego River Mission Bay	1	2015
	Silverwood Lake	3	2015
	Osoyoos Lake	2	2017
	Big Creek Lake	2	2017
	Truckee River	1	2017
